# Natural product extracts for ischemic stroke: a methodological evaluation and meta-epidemiological analysis

**DOI:** 10.3389/fphar.2025.1730699

**Published:** 2026-01-05

**Authors:** Sijin Wang, Minghui Zhang, Yao Lu, Peipei Du, Ziwen Xu, Xu Pang, Jierong Gao, Li Li, Chi Zhang

**Affiliations:** 1 Dongzhimen Hospital, Beijing University of Chinese Medicine, Beijing, China; 2 Emergency Department, Beijing Hospital of Integrated Traditional Chinese and Western Medicine, Beijing, China; 3 Institute of Basic Research in Clinical Medicine, China Academy of Chinese Medical Sciences, Beijing, China; 4 Institute for Brain Disorders, Dongzhimen Hospital, Beijing University of Chinese Medicine, Beijing, China

**Keywords:** natural product extracts, ischemic stroke, meta-epidemiological analysis, redundant systematic reviews, methodological quality

## Abstract

**Background:**

Despite numerous systematic reviews (SRs) and meta-analyses (MAs) on natural product extracts (NPEs) for ischemic stroke, their methodological limitations and redundancy are unquantified, and the impact of the Preferred Reporting Items for Systematic Reviews and Meta-Analyses (PRISMA) statement remains unclear. This meta-epidemiological study aims to evaluate the methodological quality of these SRs/MAs, examine PRISMA’s impact on the methodological quality, and quantify the frequency of redundant publications.

**Methods:**

We systematically searched for SRs/MAs of NPEs for ischemic stroke. Methodological quality was assessed with the Assessment of Multiple Systematic Reviews 2 (AMSTAR-2). Logistic regression was used to analyze associated factors. Redundancy was classified using PIC (population, intervention and comparison) frameworks as original, conceptual replication, or excessive replication. Seventy-nine SRs/MAs of NPEs were included, of which 68 focused specifically on Ginkgo biloba L. extracts (GBEs) and Panax notoginseng (Burkill) F.H.Chen extracts (PNEs) and were further analyzed for redundancy.

**Results:**

86.1% (68/79) of included SRs/MAs were rated “critically low,” primarily due to unregistered protocols (79.7%, 63/79) and absent lists of excluded studies (94.9%, 75/79). Publications following PRISMA 2020 statement showed improved protocol registration (OR = 10.04 [2.56–39.33]; p < 0.001), risk of bias assessment (OR = 22.96 [2.86–182.63]; p = 0.003), and appropriate methods for statistical combination (OR = 3.23 [1.27–8.23]; p = 0.014). Among 55 non-benchmark SRs/MAs focusing on GBEs and PNEs, 85.5% (47/55) were redundant publications, comprising 34 conceptual and 13 excessive replications.

**Conclusion:**

SRs/MAs on NPEs for ischemic stroke exhibit suboptimal methodological quality and high redundancy. Although PRISMA statement improved the methodological quality, prospective protocol registration and transparent search process are imperative to enhance evidence synthesis.

## Introduction

Stroke, the second leading cause of global mortality and primary cause of adult disability, imposes profound socioeconomic burdens (GBD, 2019 Stroke Collaborators, 2021). Despite advances in reperfusion therapies like thrombolysis and thrombectomy, significant limitations persist, including narrow treatment time windows and associated risks, leaving an urgent need for effective, accessible neuroprotective and restorative strategies. Natural product extracts (NPEs), derived from medicinal plants or animals with long histories of use, have garnered substantial interest as potential therapeutic interventions for ischemic stroke (Wu et al., 2023; Zhang et al., 2023). NPEs possessed multi-target mechanisms theorized to maintain the blood-brain barrier; antioxidation, anti-inflammation, and antiapoptosis; and promote angiogenesis (Ye et al., 2022). Importantly, these mechanisms are closely associated with the preservation and recovery of neurological function, which represents a critical outcome in stroke clinical research. Their perceived advantages, including potentially broader applicability beyond strict time windows and lower hemorrhagic risk compared to thrombolytics, have fueled a rapid surge in primary clinical research investigating their efficacy and safety (Hao et al., 2023).

Systematic reviews and meta-analyses (SRs/MAs), positioned at the apex of the evidence hierarchy, are intended to synthesize this primary data rigorously and inform clinical guidelines and practice. To enhance the transparency, completeness, and reliability of such syntheses, the Preferred Reporting Items for Systematic Reviews and Meta-Analyses (PRISMA) statement was developed as a reporting guideline (Liberati et al., 2009; Page et al., 2021). Despite the rapid proliferation of SRs/MAs evaluating NPEs and some reporting positive treatment effects (Lyu et al., 2020; Li et al., 2021; Ma et al., 2022; 2024; Zhao et al., 2024), clinical adoption remains geographically restricted. Only ginkgolides and ginkgo diterpene lactone meglumine are recommended in the Chinese guidelines for diagnosis and treatment of acute ischemic stroke 2023, representing the only two NPEs endorsed by national-level guidelines. Critically, the inclusion of these two NPEs was based on evidence from large-scale RCTs rather than high-quality SRs/MAs, which is a paradox that raises concerns about the methodological rigor of existing SRs/MAs for ischemic stroke. Moreover, the rapid increase of SRs/MAs on similar topics in this field marked by considerable redundancy (Kim and Hasford, 2020; Chapelle et al., 2021). This repetitive work wastes limited research resources and leads to the publication of numerous overlapping reviews, some of which present conflicting conclusions (Riva et al., 2018). These inconsistencies can confuse clinical decision-makers, encourage selective citation of evidence, and weaken overall confidence in systematic reviews as a reliable source of evidence (Puljak and Lund, 2023).

However, the methodological quality and prevalence of overlapping publications specifically addressing NPEs for ischemic stroke remain poorly characterized. Prior overviews in this field have primarily focused on assessing methodological quality for a single class of interventions, leaving the extent and drivers of redundancy unquantified (Tian et al., 2021; Meng et al., 2024). Addressing these gaps is essential to evaluate the reliability of high-level evidence supporting NPEs and to steer future evidence synthesis toward greater rigor, clinical relevance, and impact on patient care. Consequently, this meta-epidemiological study aims to: (1) evaluate the methodological quality of SRs/MAs on NPEs for ischemic stroke; (2) examine the impact of the PRISMA 2020 statement on the methodological quality; and (3) quantify the frequency of redundant publications.

## Methods

### Inclusion and exclusion criteria

This study included SRs/MAs meeting the following criteria: (1) Study Design: SRs/MAs only included randomized controlled trials (RCTs). (2) Population: Included RCTs enrolled patients with a clinically confirmed diagnosis of ischemic stroke. (3) Intervention: Included RCTs investigated an intervention defined as NPEs, irrespective of its dosage form or formulation. (4) Comparator: Included RCTs used guideline-recommended conventional therapy (with or without additional therapies) as the control group. (5) Outcomes: SRs/MAs reporting at least one functional outcome were eligible for inclusion.

Exclusion Criteria including: (1) overview of SRs; (2) umbrella reviews; (3) network Meta-Analyses; (4) individual patient data Meta-Analyses; (5) preclinical studies; (6) protocol; (7) publications where the full text was unavailable; (8) duplicate publication.

### Search strategy

The literature search was conducted in two sequential phases.

### Phase 1: initial systematic search

An initial systematic literature search was conducted in MEDLINE via PubMed to identify SRs/MAs of RCTs investigating NPEs for ischemic stroke. The search encompassed records from database inception until 29 May 2025, which was the date of the final search, ensuring the inclusion of the most current evidence available at the time of our study. The search strategy employed specific compound names and proprietary drug names of NPEs as keywords to retrieve SRs/MAs of ischemic stroke. No language restrictions were applied.

### Phase 2: supplemental search of regional databases

Based on the findings above, we identified the most frequently investigated NPEs, defined as those with more than three published SRs/MAs retrieved from PubMed. Based on the findings from this initial phase, which identified the most frequently investigated NPEs, we conducted a supplemental search in regional databases. This targeted search, using equivalent strategies, aimed to ensure a comprehensive capture of literature and enable a specific analysis of redundancy for these prominent NPEs. It encompassed records from database inception until 10 June 2025. All records from PubMed and regional databases were subjected to identical eligibility assessment and screening methodology.

No language restrictions were applied. The search strategy was built using free-text terms in the title and abstract fields, combined with Boolean operators. The search keywords included “systematic review,” “ischemic stroke,” and the names of commonly studied natural product extracts—encompassing both compound names and proprietary drug names—along with derivative terms of these keywords. The complete search string used for PubMed is presented below:

(systematic review [Title/Abstract] OR meta analysis [Title/Abstract])) AND (ischemic stroke [Title/Abstract] OR cerebral infarction [Title/Abstract] OR cerebral ischemia [Title/Abstract] OR acute ischemic stroke [Title/Abstract]) AND ((ginkgo [Title/Abstract] OR ginkgolide [Title/Abstract] OR “ginkgo ketone ester” [Title/Abstract] OR “ginkgo diterpene lactone*” [Title/Abstract] OR Shuxuening [Title/Abstract] OR danhong [Title/Abstract] OR Salvia [Title/Abstract] OR “Salvia miltiorrhiza” [Title/Abstract] OR safflower [Title/Abstract] OR “Carthamus tinctorius” [Title/Abstract] OR sanqi [Title/Abstract] OR “Panax notoginseng” [Title/Abstract] OR “Panax notoginseng saponins” [Title/Abstract] OR xuesaitong [Title/Abstract] OR “Ixeris sonchifolia” [Title/Abstract] OR kudiezi [Title/Abstract] OR dengzhan [Title/Abstract] OR Breviscapine [Title/Abstract] OR “Erigeron breviscapus” [Title/Abstract] OR Puerarin [Title/Abstract] OR “Pueraria lobata” [Title/Abstract] OR chuanxiong [Title/Abstract] OR chuanxiongqin [Title/Abstract] OR Tetramethylpyrazine [Title/Abstract] OR shenxiong [Title/Abstract] OR shenmai [Title/Abstract] OR shengmai [Title/Abstract] OR Gastrodin [Title/Abstract] OR shuxuetong [Title/Abstract] OR Coniferin [Title/Abstract] OR Quercetin [Title/Abstract] OR Epigallocatechin gallate [Title/Abstract] OR Resveratrol [Title/Abstract] OR Apocynin [Title/Abstract] OR Baicalin [Title/Abstract] OR Naringin [Title/Abstract] OR Triptolide [Title/Abstract] OR Phycocyanobilin [Title/Abstract]))

The search strategies for regional databases were adapted based on the PubMed search results. We refined the strategy to include only the most frequently investigated NPEs identified through the PubMed search. The search string for CNKI is provided as an example below:

TKA=(‘系统综述’+‘系统评价’+‘meta’) AND TKA=(‘银杏’+‘金纳多’+‘三七总皂苷’+‘舒血宁’+‘血塞通’+‘三七’+‘血栓通’) AND TKA=(‘脑梗死’+‘脑梗’+‘腔隙性脑梗’+ ‘腔梗’+‘脑栓塞’+‘脑血栓’+‘脑卒中’+‘卒中’+‘中风’+ ‘脑缺血’+‘脑血管病’+ ‘缺血性脑中风’ +‘缺血性脑卒中’+‘缺血性中风’+‘缺血性卒中’)

The complete search strategy of is provided in Supplementary Material S1.

### Study selection and data extraction

Two authors (WSJ and ZMH) independently checked each paper in full and extracted data in duplicate using a standardized form to ensure consistency of extracted data for each study. Disagreements were resolved through discussions. The following data were systematically extracted from each included SRs/MAs: (1) Study characteristics: publication year, first author, funding sources, and publication language; (2) PICO elements: study design, population characteristics, intervention and comparator details, and outcome measures; (3) Protocol registration status; (4) Citation of prior SRs/MAs on identical topics; (5) Included RCTs.

### Methodological quality assessment

Two authors (WSJ and ZMH) separately assessed the quality of included SRs/MAs using the Assessment of Multiple Systematic Reviews 2 (AMSTAR-2). Any discrepancies in the 16 items were resolved by another author (DPP). Each of the 16 items was rated as “yes” (if the item was answered completely), “no” (the item was absent or not appropriate), or “partial yes” (some of the subitems incomplete). An overall rating (high, moderate, low, and critically low) was evaluated as follows: overall quality was assessed as high when there was no or just one noncritical weakness (item 2, item 4, item 7, item 9, item 11, item 13, and item 15 as critical items; others as noncritical items); moderate when there was just more than one noncritical weakness; low when there was just one critical flaw; and critically low when there was more than one critical flaw.

### Definition of redundancy

SRs/MAs were categorized based on their PIC elements (Population, Intervention, and Comparison) and whether they justified conducting a review on an existing topic. As the inclusion criteria for SRs/MAs were restricted to those reporting functional outcomes, outcome measures were not incorporated as elements in the redundancy assessment. Reviews sharing identical PIC frameworks were classified as replication publications. The first published SR/MA addressing a specific PIC was identified as the benchmark. Subsequent publications sharing identical PIC frameworks were systematically screened. Non-benchmark SRs/MAs was assigned to one of three redundancy categories adapted from Tugwell et al. (2020): (1) Original:, subsequent updates by the same research team, or SRs/MAs explicitly citing prior reviews and justified the new analysis; (2) Conceptual replication: SRs/MAs modifying the original PIC, including adding new RCTs, focusing on subgroups, without providing justification for duplicating the core research question; (3) Excessive replication: SRs/MAs with PIC identical to existing benchmarks that lacked scientific rationale for duplication and failed to provide justification for replicating the core research question. Conceptual replication and excessive replication were identified as redundant SRs/MAs. The classification was performed independently by two authors (WSJ, PX) using predefined decision rules derived from the Tugwell framework. Disagreements between reviewers were resolved by consensus.

### Statistical analysis

We conducted a descriptive analysis of the methodological quality of the included SRs/MAs. Prespecified factors–publication before or after PRISMA 2020 statement–were analyzed using logistic regression analyses. For SRs/MAs addressing identical research questions, we quantified the total number of such reviews and the extent of redundancy, categorizing redundant studies as either conceptual replication or excessive replication. The inter-rater agreement prior to consensus was assessed using Cohen’s kappa statistic. Subsequently, we calculated coverage probabilities for RCTs within SRs/MAs on the same topic. The coverage probability for a SR/MA was defined as the ratio of the number of RCTs included to the total number of relevant RCTs published before that specific SRs/MAs’ publication year. All statistical analyses were performed using R software (version 4.3.2), with statistical significance defined as a two-tailed p-value <0.05.

## Results

### Search results

The Phase 1 literature search identified 79 unique records for screening. After screening titles, abstracts, and subsequently full texts, 46 records were excluded with reasons, including overviews of SRs, umbrella reviews, network meta-analyses, preclinical studies, unretrievable full texts, or PICO inconsistencies, resulting in 33 SRs/MAs being included. These 33 reviews investigated a total of 10 distinct NPEs. The most frequently evaluated NPEs were *Ginkgo biloba L.* extracts (GBEs), accounting for 42.4% (14/33) of the included SRs/MAs, followed by *Panax notoginseng (Burkill) F.H.Chen* extracts (PNEs), which constituted 24.2% (8/33).

Based on these findings from Phase 1, a supplemental search was conducted in regional databases specifically targeting GBEs and PNEs. This search identified 357 records. After removing 150 duplicates, 207 records were screened. Following the assessment of titles, abstracts, and full texts, 46 records were excluded using the same eligibility criteria as in Phase 1, yielding 49 additional SRs/MAs for inclusion.

**FIGURE 1:**
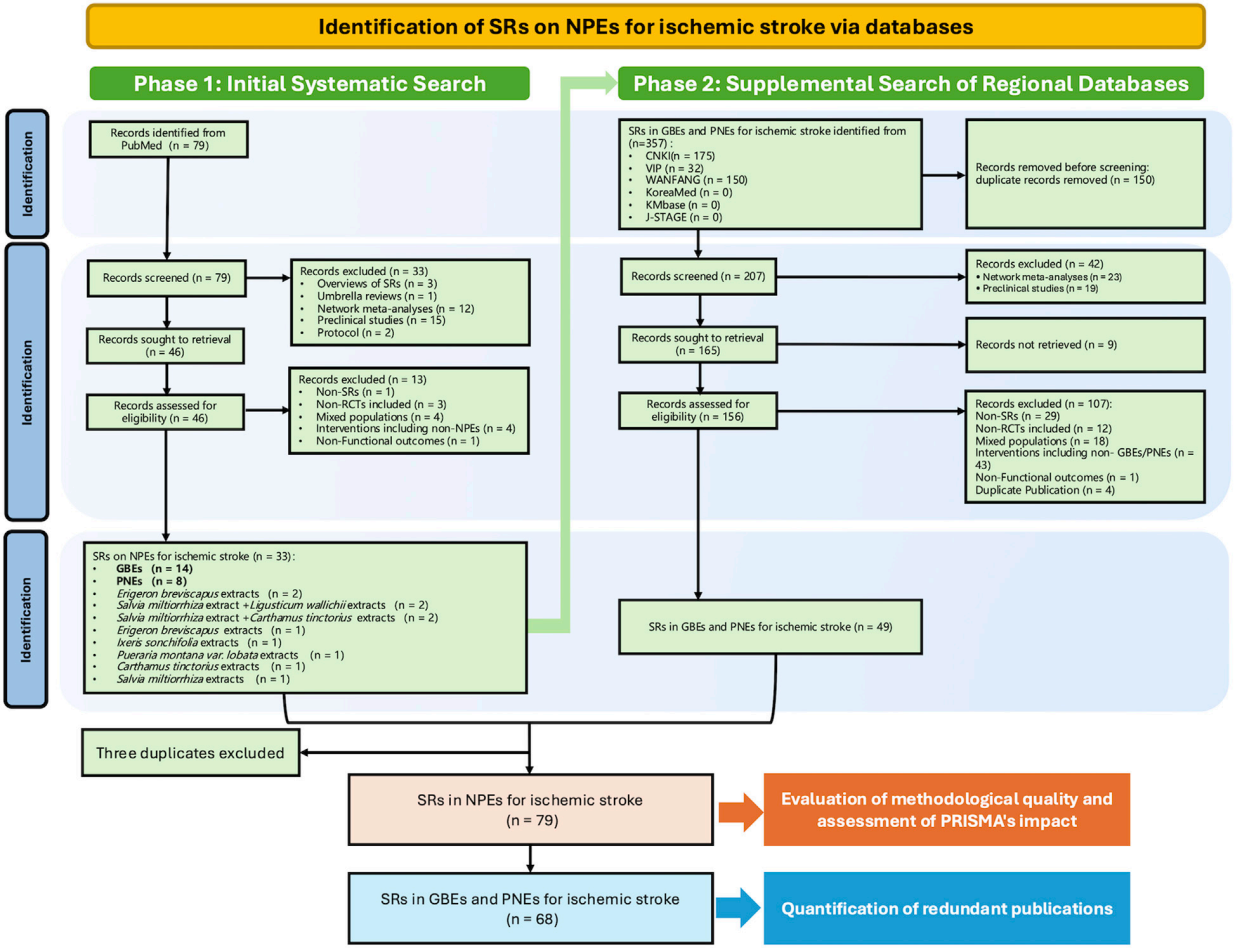
Flow diagram of the study selection process. Abbreviations: SR, systematic review; RCT, randomised controlled trial; NPE, Natural Product Extract; GBE, Ginkgo biloba L. extract; PNE, Panax notoginseng (Burkill) F.H.Chen extract; CNKI: China National Knowledge Infrastructure (https://www.cnki.net/); VIP: China Science and Technology Journal Database (http://www.cqvip.com/); WANFANG: Wanfang Data Knowledge Service Platform (http://www.wanfangdata.com.cn/); KoreaMed: Korean Medical Database (https://koreamed.org/); KMbase: Korean Medical Database (https://kmbase.medric.or.kr/); J-STAGE: Japan Science and Technology Information Aggregator, Electronic (https://www.jstage.jst.go.jp/).

Combining the results from both phases and removing 3 duplicate publications identified across the datasets, a total of 79 SRs/MAs on NPEs were included for the methodological quality assessment. Of these, the 68 SRs/MAs focusing specifically on GBEs and PNEs were further analyzed for the quantification of redundant publications. The complete study selection process is detailed in the PRISMA flow diagram (Figure 1). The complete list of retrieved records and their respective reasons for exclusion are provided in Supplementary Material S2.

### Study characteristics

Table 1 summarizes the characteristics of the 79 included studies. Of these, 65.8% (52/79) were published in Chinese, compared with 34.2% (27/79) in English. In terms of publication timeline, 59.5% (47/79) appeared in or before 2020, while 40.5% (32/79) were published thereafter. Protocols were registered in 20.3% (16/79) of the studies, and 57.0% (45/79) disclosed conflicts of interest.

**TABLE 1:** Characteristics of included 79 SRs/MAs in this study.

Characteristics	All SRs/MAs, n (%)
Type of medicine	
Ginkgo biloba L. extracts	34 (43.0)
Panax notoginseng (Burkill) F.H.Chen extracts	34 (43.0)
Erigeron breviscapus extracts	2 (2.5)
Salvia miltiorrhiza extracts + Ligusticum wallichii extracts	2 (2.5)
Salvia miltiorrhiza extracts + Carthamus tinctorius extracts	2 (2.5)
Erigeron breviscapus extracts	1 (1.3)
Ixeris sonchifolia extracts	1 (1.3)
Pueraria montana var. lobata extracts	1 (1.3)
Carthamus tinctorius extracts	1 (1.3)
Salvia miltiorrhiza extracts	1 (1.3)
Publication language	
English	27 (34.2)
Chinese	52 (65.8)
Publication year	
Until 2020 inclusive	47 (59.5)
After 2020	32 (40.5)
Conflict of interest	
Yes	0 (0.0)
No	34 (43.0)
Not reported	45 (57.0)
Protocol registration	
Yes	16 (20.3)
No	63 (79.7)

Abbreviations: SR/MA, systematic review and meta-analysis.

### Methodological quality assessment

**FIGURE 2:**
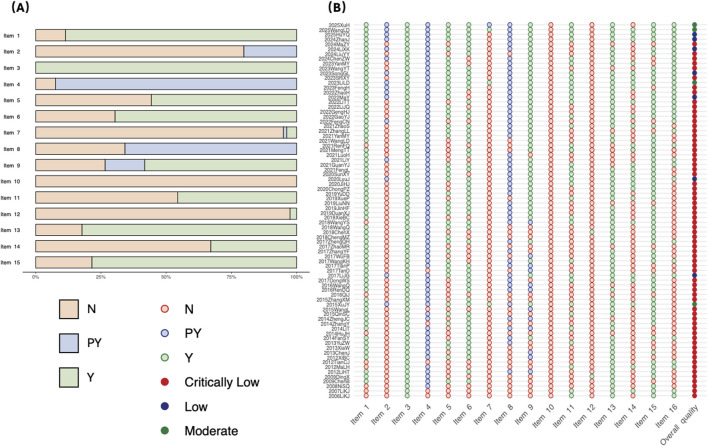
Methodological quality assessment of included 79 SRs/MAs by AMSTAR 2. **(A)** The percentages of the judgments of the authors about each item. **(B)** Each item of the judgments of the authors. Abbreviations: AMSTAR 2, A MeaSurement Tool to Assess systematic Reviews 2; Y, Yes; PY, Partial Yes; N, No; NA, Not Applicable. Item 1: Did the research questions and inclusion criteria for the review include the components of PICO?; Item 2: Did the report of the review contain an explicit statement that the review methods were established prior to the conduct of the review, and did the report justify any significant deviations from the protocol?; Item 3: Did the review authors explain their selection of the study designs for inclusion in the review?; Item 4: Did the review authors use a comprehensive literature search strategy?; Item 5: Did the review authors perform study selection in duplicate?; Item 6: Did the review authors perform data extraction in duplicate?; Item 7: Did the review authors provide a list of excluded studies and justify the exclusions?; Item 8: Did the review authors describe the included studies in adequate detail?; Item 9: Did the review authors use a satisfactory technique for assessing the risk of bias?; Item 10: Did the review authors report on the sources of funding?; Item 11: Did the review authors use appropriate methods for the statistical combination of results?; Item 12: Did the review authors assess the potential impact of RoB in individual studies on the results?; Item 13: Did the review authors account for RoB in individual studies when interpreting/discussing the results of the review?; Item 14: Did the review authors provide a satisfactory explanation for, and discussion of, any heterogeneity?; Item 15: Did the review authors carry out an adequate investigation of publication bias?; Item 16: Did the review authors report any potential sources of conflict of interest?.

Figure 2 illustrates the detailed assessment of each AMSTAR-2 item using a traffic light plot and summarizes the overall quality ratings in a stacked bar chart. The overall quality was rated as critically low for 86.1% (68/79) of SRs/MAs. Prevalent critical deficiencies included the absence of protocol registration (79.7%, Item 2), missing lists of excluded studies (94.9%, Item 7), and inappropriate methods for statistical combination, primarily due to a lack of investigation into heterogeneity sources (54.4%, Item 11). Among non-critical items, inadequate description of PICOS elements (34.1%, Item 8) and omission of conflict of interest declarations (57.0%, Item 16) were notable.

A stratified analysis focusing on the 68″critically low” rated reviews was performed to identify the most influential deficiencies (Supplementary Material Table S2). This analysis revealed that shortcomings in Items 11 (appropriate methods for statistical combination), 14 (explanation for any heterogeneity), and 16 (conflict of interest declaration) were the most prevalent drivers of the low ratings. While these findings should be interpreted with caution due to sample size constraints, they suggest that within the specific context of SRs/MAs on NPEs for stroke, methodologies for handling heterogeneity and ensuring reporting transparency are most strongly linked to the final quality assessments.

### PRISMA statement and methodological quality

Table 2 presents associations between PRISMA statement and included SRs/MAs’ methodological quality. Post-PRISMA statement publications demonstrated significantly higher rate of protocol registration (OR = 10.04; 95% CI: 2.56–39.33; p < 0.001) (Item 2), RoB assessment (OR = 22.96; 95% CI: 2.89–182.63; p = 0.003) (Item 9), and appropriate methods for statistical combination (OR = 3.23; 95% CI: 1.27–8.23; p = 0.014) (Item 11).

**TABLE 2:** Associated between the publication of PRISMA 2020 and critical items of AMSTAR-2.

Item	N	Y/PY/NA	OR	P value
Item 2	(N = 63)	(N = 16)		
Until 2020 inclusive	44 (69.8%)	3 (18.8%)	10.04 (2.56–39.33)	p < 0.001*
After 2020	19 (30.2%)	13 (81.2%)		
Item4	(N = 6)	(N = 73)		
Until 2020 inclusive	6 (100%)	41 (56.2%)	—	—
After 2020	0 (0%)	32 (43.8%)		
Item7	(N = 75)	(N = 4)		
Until 2020 inclusive	46 (61.3%)	1 (25%)	4.76 (0.47–47.96)	p = 0.186
After 2020	29 (38.7%)	3 (75%)		
Item9	(N = 21)	(N = 58)		
Until 2020 inclusive	20 (95.2%)	27 (46.6%)	22.96 (2.89–182.63)	p = 0.003*
After 2020	1 (4.8%)	31 (53.4%)		
Item11	(N = 43)	(N = 36)		
Until 2020 inclusive	31 (72.1%)	16 (44.4%)	3.23 (1.27–8.23)	p = 0.014*
After 2020	12 (27.9%)	20 (55.6%)		
item13	(N = 14)	(N = 65)		
Until 2020 inclusive	6 (42.9%)	41 (63.1%)	0.44 (0.14–1.42)	p = 0.169
After 2020	8 (57.1%)	24 (36.9%)		
Item15	(N = 17)	(N = 62)		
Until 2020 inclusive	11 (64.7%)	36 (58.1%)	1.32 (0.43–4.04)	p = 0.622
After 2020	6 (35.3%)	26 (41.9%)		

Abbreviations: PRISMA, the Preferred Reporting Items for Systematic Reviews and Meta-Analyses; OR, odds ratio; Y, yes; PY, partial yes; N, no; NA, Not Applicable. Item 2: Did the report of the review contain an explicit statement that the review methods were established prior to the conduct of the review, and did the report justify any significant deviations from the protocol?; Item 4: Did the review authors use a comprehensive literature search strategy?; Item 7: Did the review authors provide a list of excluded studies and justify the exclusions?; Item 9: Did the review authors use a satisfactory technique for assessing the risk of bias?; Item 11: Did the review authors use appropriate methods for the statistical combination of results?; Item 13: Did the review authors account for RoB in individual studies when interpreting/discussing the results of the review?; Item 15: Did the review authors carry out an adequate investigation of publication bias?

### Number of redundant SRs/MAs

Table 3 and Supplementary Figure S1 show descriptive statistics of all 13 NPEs topics. Of the 68 SRs/MAs focusing on GBEs and PNEs, 13 were classified as benchmark SRs/MAs, and the remaining 55 were evaluated for redundancy. The Cohen’s kappa statistic for the initial redundancy classification was 0.852. The individual ratings and the final consensus classification for each SRs were detailed in Supplementary Material S3. Among these, 14.5% (8/55) were original (7 SRs/MAs providing adequate justification for replicating existing research and 1 SRs/MAs was defined as update SR) and 85.5% (47/55) demonstrated duplication: 34/55 were classified as conceptual replications and 13/55 as excessive replications, with none. Temporal clustering was particularly pronounced for specific interventions: four SRs/MAs on Xuesaitong soft capsules published in 2022, with none of them registered, three SRs/MAs of multi-*Ginkgo biloba* extracts clustered in 2015, and five *Ginkgo diterpene lactone meglumine* injection SRs/MAs published between 2021 and 2025.

**TABLE 3:** Redundant rate and coverage of included RCTs of 68 included SRs/MAs on GBEs and PNE**s**.

Product name	Dosage form	Active component(s)	Redundant rate N (%)	Coverage of included RCTs (range)
*Panax notoginseng* (Burkill) F.H.Chen extracts				
Sanqitongshu	Capsule	*Panax notoginseng* saponins	1 (100.0%)	62.5%–64.3%
Xuesaitong	Soft capsule	*Panax notoginseng* saponins	6 (100.0%)	17.8%–40.0%
Xuesaitong	Injection	*Panax notoginseng* saponins	8 (72.7%)	6.7%–19.7%
Xueshuantong	Injection	*Panax notoginseng* saponins	6 (75.0%)	4.9%–36.1%
Multi-*Panax notoginseng* Preparations	Mixed forms	*Panax notoginseng* saponins	3 (50.0%)	8.1%–63.9%
Ginkgo biloba L. extracts				
Shuxuening	Injection	Total flavonoid glycosides	3 (75.0%)	7.5%–71.5%
Yinxingdamo	Injection	Total flavonoid glycosides + Dipyridamole	3 (100.0%)	51.9%–73.1%
Ginkgo diterpene lactone meglumine	Injection	Ginkgolides (A/B/K)	6 (100.0%)	8.2%–41.0%
Ginkgolide injection	Injection	Bilobalide + Ginkgolides (A/B/C)	1 (100.0%)	33.3%–100.0%
Ginkgo ketone esters	Mixed forms	Ginkgo ketone esters	0 (0.0%)	100%
Ginkgo biloba leaf tablets	Tablet	Ginkgo biloba extract	1 (100.0%)	31.3%–100.0%
Ginkgo biloba leaf extract	Injection	Ginkgo biloba extract	1 (100.0%)	48.6%–57.6%
Multi-ginkgo biloba preparations	Mixed forms	Ginkgo biloba extract	8 (80.0%)	6.0%–34.1%

Abbreviations: RCT, randomized controlled trial; SRs/MAs, systematic reviews and meta-analyses; GBEs, *Ginkgo biloba L*. extracts; PNEs, *Panax notoginseng (Burkill) F.H.Chen* extracts.

### Coverage rate of included RCTs

Three studies (2006LiKJ; 2008NiSQ; 2016RenDQ) failed to provide reference lists or supplementary attachments, precluding traceability of the original RCTs they included. Among traceable SRs/MAs, the coverage probabilities for eligible RCTs ranging widely from 6.7% to 100.0% with 86.7% (59/68) of these demonstrating coverage probabilities of below half (Supplementary Material Figure S). These patterns of omission and selective inclusion were observed across nearly all therapeutic topics. The coverage probabilities for ‘Xuesaitong Injection’ and ‘Xueshuantong Injection’ ranged from 6.7% to 19.7% and 8.1%–63.9%, respectively (Supplementary Material Table S1). Notably, substantial heterogeneity in trial inclusion persisted even when PIC frameworks were identical in their specific details, including restrictions on time-from-onset and well-defined population inclusion criteria, as exemplified by the marked divergence in coverage rates between 2007LiKJ (63.9%) and 2009ChenB (23.1%).

## Discussion

Our meta-epidemiological study revealed low methodological quality and high redundancy in nearly ninety percent of SRs/MAs on NPEs for ischemic stroke. These SRs/MAs also exhibited substantial heterogeneity in included primary studies across reviews on the same topic. Notably, post-PRISMA publications showed methodological improvements, particularly in protocol registration, RoB assessment and statistical combination.

The pervasive issues identified critically contribute to unreliable conclusions and resource waste, ultimately impeding the translation of NPEs into clinical practice. Low-quality SRs/MAs fail to provide reliable evidence for regulatory agencies or guideline committees, delaying regulatory approval of promising NPEs identified in preclinical studies. These problems are driven by a complex interplay of factors, including the academic incentive structure that prioritizes quantity over quality, language barriers, and institutional evaluation systems that inadequately reward rigorous systematic reviewing. This methodological crisis is near-universal in natural products’ SRs/MAs. Our findings align with several overviews demonstrating that more than 85% of SRs/MAs on natural products for ischemic stroke are rated “critically low” on AMSTAR-2 (Tian et al., 2021; Meng et al., 2024). Low quality persists beyond stroke interventions, as evidenced by the finding that 12 out of 14 SRs/MAs on Chinese herbal medicine for psoriasis scored “critically low” (Zhang et al., 2021). Moreover, redundant and methodologically flawed reviews waste scarce resources that could fund essential research. This inefficiency mirrors the “evidence waste” identified in global research synthesis. Such redundancy is widespread, documented across numerous diseases and interventions (Katsura et al., 2021; Mendoza et al., 2021; McDonald et al., 2022). As demonstrated in research evaluating redundant studies in the health field, nearly 50% of scholarly resources were expended on redundant SRs/MAs(Lund et al., 2022).

These issues of low quality and redundancy manifest as unreliable conclusions, which may include overestimated treatment effects or contradictory findings. On the one hand, the evidence is often characterized by an overrepresentation of positive outcomes. Such reviews may also misguide future research priorities by selectively overrepresenting positive trials. Findings from a study on citation bias in otolaryngology demonstrate that summary effects in these SRs/MAs may be biased toward statistically significant findings (Vassar et al., 2021). Furthermore, most SRs/MAs included in our study exclusively searched published literature, potentially exacerbating publication bias favoring positive results. Compounding this issue, nearly thirty percent of included SRs/MAs either omitted publication bias assessment or included fewer than 10 primary studies, significantly increasing the likelihood of overestimating treatment effects. On the other hand, extreme variability in RCT coverage directly also fuels conflicting conclusions across SRs/MAs on identical topics (Helfer et al., 2015; Hacke and Nunan, 2020). This issue is illustratively demonstrated in surgical SRs/MAs, where overlapping reviews reached contradictory conclusions despite shared objectives (Katsura et al., 2021). Consequently, this inconsistency, amplified by unregistered protocols and unreported exclusion lists, compromises evidence reliability and exemplifies the scientific futility of redundant reviews.

Encouragingly, observed post-PRISMA 2020 enhancements in protocol registration, RoB assessment, and appropriate methods for the statistical combination underscore the capacity of standardized reporting tools to elevate methodological rigor. Although these positive associations should be interpreted with caution due to the limited number of studies, which resulted in relatively wide confidence intervals, they nevertheless provide a robust and encouraging signal of improvement. It is important to note that these observed improvements are likely multifactorial, coinciding with increased methodological awareness, stricter editorial policies, and the broader adoption of reporting guidelines, of which PRISMA is one key component. Consistent evidence demonstrates PRISMA’s positive impact on SR quality within the natural products field. An analysis examining the impact of the PRISMA statement revealed significant improvements in the methodological quality of systematic reviews of traditional Chinese medicine for ischemic stroke, specifically in the reporting of inclusion/exclusion criteria and risk of bias assessment, which mandates transparent reporting of these items (Sun et al., 2023). A cross-sectional study, focusing on evaluating the methodological quality of SRs of traditional Chinese medicine, also indicated slightly better performance in conducting comprehensive literature searches among SRs/MAs published with PRISMA statement (Chung et al., 2015).

Eighty-five percent of post-benchmark SRs/MAs in our study were redundant, alarmingly without citations to prior reviews. The primary drivers of redundancy include failure to systematically retrieve existing evidence and lack of protocol registration. These systemic deficiencies are pervasive in redundant SRs/MAs. For example, a meta-epidemiological study on pharmacological interventions reported low citation rates of prior SRs/MAs, with only one-fifth discussed previous findings ^25^. This reflects inadequate study planning, indicating that authors frequently omit comprehensive literature searches and that existing evidence syntheses remain incomplete (Helfer et al., 2015; Créquit et al., 2016). Lack of protocol registration further accelerates redundancy, manifested as clusters of publications on identical topics within short timeframes. For instance, four SRs/MAs on Xuesaitong Soft Capsules for ischemic stroke were published in 2022, none of them was registered. Similar clusters were observed in Xuesaitong Injection and Ginkgolide Injection. This pattern was also documented in COVID-19 research (McDonald et al., 2022). These observations collectively indicate that investigators of SRs/MAs need to carefully examine whether SRs/MAs on the same topic already exist, either published or registered, prior to initiating new reviews.

To systematically address these challenges, we propose concrete solutions targeting different stages of evidence synthesis. First, mandatory prospective registration in open platforms should be enforced by journals and funding agencies, with automated checks for topic overlap at submission. Second, AI-driven meta-synthesis tools should be developed to automate systematic searching, update evidence maps in real-time, and alert researchers to existing reviews, thereby preventing unintentional duplication. For NPEs evidence regulation and global integration, our findings underscore the urgent need for standardized methodological requirements. Regulatory agencies and guideline committees should formally mandate high AMSTAR-2 ratings and proof of non-redundancy as prerequisites for considering evidence from systematic reviews.

This study provides a significant advance beyond prior overviews in this field, which were typically confined to evaluating methodological quality for a specific drug class. By concurrently assessing methodological rigor, quantitatively examining the impact of PRISMA, and systematically quantifying and dissecting the drivers of redundancy, our analysis offers a comprehensive understanding of the crises impairing evidence synthesis for NPEs.

While our study represents the first comprehensive evaluation of both methodological quality and redundancy specifically for SRs/MAs of NPEs, key limitations warrant consideration. First, the exclusion of advanced synthesis methods including network meta-analyses and overviews of SRs/MAs, while necessary for methodological homogeneity, may introduce selection bias and limit the generalizability of our findings to the entire evidence landscape of NPEs. Secondly, though necessary for methodological homogeneity, the exclusion of advanced synthesis methods, including network meta-analysis and individual participant data meta-analysis, limits insights into comprehensive evidence landscapes. Third, by basing redundancy on PIC elements without considering outcomes, we may have overestimated conceptual replication, as reviews using different functional scales could address distinct clinical questions. This limitation should be considered when interpreting our findings. Fourth, the high proportion of included studies published in Chinese introduces a potential for language bias. Moreover, while our classification of review redundancy was guided by a well-established operational framework derived from expert consensus (Chapelle et al., 2023), the general absence of an authoritative, field-standardized definition for ‘redundant systematic reviews’ must be acknowledged. This lack of consensus introduces a potential element of methodological subjectivity, and our findings should be interpreted considering this conceptual limitation.

Our findings yield critical imperatives for enhancing evidence synthesis. To ensure methodological rigor, authors and journals must strictly enforce PRISMA reporting during SRs/MAs writing or review and implement standardized quality assessment using tools like AMSTAR-2. To combat redundancy and minimize waste, researchers should consult trial registries and existing SRs/MAs before initiating new studies and explain how they consider the necessity of research (Chalmers et al., 2014), while journals mandate protocol registration and conduct redundancy screening via PICOS compliance checks. To transform the evidence ecosystem, future initiatives will establish an AI-driven evidence synthesis platform that will be developed for automated SR generation and real-time evidence mapping of NPEs. A shared RCT repository will be created to standardize primary data and resolve coverage inconsistencies across SRs/MAs. These integrated initiatives, featuring protocol preregistration, methodological quality tracking, will systematically prevent redundant research, accelerate evidence translation, and reduce waste in NPE through continuous evidence integration.

## Conclusion

Current SRs/MAs evaluating NPEs for ischemic stroke demonstrate critically deficient methodological rigor and evidence redundancy. Although the publication of the PRISMA statement has contributed to modest improvements in certain methodological aspects, substantial challenges persist. To enhance the reliability and utility of future evidence syntheses, strict adherence to prospective protocol registration and the implementation of fully transparent search processes are imperative.

## Data Availability

The original contributions presented in the study are included in the article/Supplementary Material, further inquiries can be directed to the corresponding authors.
