# Supplementary material for: Natural product extracts for ischemic stroke: a methodological evaluation and meta-epidemiological analysis
Source: Front Pharmacol. 2026 Jan 5;16:1730699. doi: 10.3389/fphar.2025.1730699 (PMC12813109; doi:10.3389/fphar.2025.1730699)
Supplement: Supplementary file 1 [file Table1.docx]

**Table S1 Descriptive statistics of 13 included NPEs topics**

| **Product Name** | **Dosage Form** | **ID** | **Coverage** | **Active Component(s)** | **Overlapping** |
| --- | --- | --- | --- | --- | --- |
|  |  |  |  |  | **n（%）** |
| **Panax notoginseng Preparations** |  |  |  |  |  |
| Sanqitongshu | Capsule | 2013XiaW | 64.3%(9/14) | Panax notoginseng saponins | 1(100.0%) |
|  |  | 2018ChenX | 62.5%(10/16) |  |  |
| Xuesaitong | Soft capsule | 2022GaoYJ | 40.0%(18/45) | Panax notoginseng saponins | 6(100.0%) |
|  |  | 2022GengHJ | 17.8%（8/45） |  |  |
|  |  | 2022FengCN | 35.6%（16/45） |  |  |
|  |  | 2022LiJQ | 26.7%（12/45） |  |  |
|  |  | 2024LiXK | 30.4%（14/46） |  |  |
| Xuesaitong | Injection | 2009DingX | 15.6%（12/77） | Panax notoginseng saponins | 8(72.7%) |
|  |  | 2012TianCJ | 18.3%（19/104） |  |  |
|  |  | 2014ZhangY | 18.6%（21/113） |  |  |
|  |  | 2014ZhengCJ | 11.5%（13/113） |  |  |
|  |  | 2015ZhangXM | 19.7%（23/117） |  |  |
|  |  | 2017TianP | 8.6%（11/128） |  |  |
|  |  | 2019DuanXJ | 16.1%（23/143） |  |  |
|  |  | 2019LiuNN | 7.7%（11/143） |  |  |
|  |  | 2020SunXY | 6.7%（10/150） |  |  |
|  |  | 2021FengL | 8.0%（12/150） |  |  |
|  |  | 2021YanMY | 6.7%（10/150） |  |  |
|  |  | 2023FengH | 13.2%（20/151） |  |  |
| Xueshuantong | Injection | 2012LiHT | 20.2%（17/84） | Panax notoginseng saponins | 6(75.0%) |
|  |  | 2013ChenJ | 14.4%（13/90） |  |  |
|  |  | 2016WangQ | 22.8%（31/136） |  |  |
|  |  | 2017WuFB | 4.9%（7/143） |  |  |
|  |  | 2017ZhaoMR | 9.8%（14/143） |  |  |
|  |  | 2018ChengMZ | 20.4%（30/147） |  |  |
|  |  | 2021RenFQ | 12.1%（19/157） |  |  |
|  |  | 2023SongGL | 36.1%（57/158） |  |  |
| Multi-Panax notoginseng Preparations | Mixed forms | 2007LiKJ | 63.9%（23/36） | Panax notoginseng saponins | 3(50.0%) |
|  |  | 2009ChenB | 23.1%（9/39） |  |  |
|  |  | 2016QiJ | 27.3%（24/88） |  |  |
|  |  | 2021WangLD | 29.5%（43/146） |  |  |
|  |  | 2023WangYT | 8.1%（13/160） |  |  |
|  |  | 2023ShiXY | 26.3%（42/160） |  |  |
|  |  | 2024LiuYY | 12.3%（20/162） |  |  |
| **Ginkgo biloba Preparations** |  |  |  |  |  |
| Shuxuening | Injection | 2012XiBC | 7.5%(8/106) | Total flavonoid glycosides | 3(75.0%) |
|  |  | 2014HuJH | 14.6%(19/130) |  |  |
|  |  | 2014LiT | 13.1%(17/130) |  |  |
|  |  | 2023LiLD | 9.1%(15/165) |  |  |
|  |  | 2024ZhanJ | 71.5%(118/165) |  |  |
| Yinxingdamo | Injection | 2008NiSQ | NA | Total flavonoid glycosides+Dipyridamole | 3(100.0%) |
|  |  | 2013YuZW | 51.9%(14/27) |  |  |
|  |  | 2016RenDQ | NA |  |  |
|  |  | 2019XueP | 73.1%(38/52) |  |  |
| Ginkgo diterpene lactone meglumine | Injection | 2018WangQ | 41.0%(16/39) | Ginkgolides (A/B/K) | 6(100.0%) |
|  |  | 2019JinFH | 15.8%(9/57) |  |  |
|  |  | 2021ZhangLL | 30.2%(26/86) |  |  |
|  |  | 2022ZhaoH | 27.2%(25/92) |  |  |
|  |  | 2023YanMY | 17.7%(17/96) |  |  |
|  |  | 2025WangLD | 8.2%(8/98) |  |  |
|  |  | 2025XuH | 25.5%(25/98) |  |  |
| Ginkgolide | Injection | 2021LuoH | 33.3%(8/24) | Bilobalide+Ginkgolides (A/B/C) | 1(100.0%) |
|  |  | 2021MengTT | 100.0%(24/24) |  |  |
| Ginkgo ketone esters | Mixed forms | 2021GuanYJ | 100.0%(16/16) | Ginkgo ketone esters | 0(0.0%) |
| Ginkgo biloba leaf tablets | Tablet | 2021MengTT | 31.3%(10/32) | Ginkgo biloba extract | 1(100.0%) |
|  |  | 2024ChenZW | 100.0%(32/32) |  |  |
| Ginkgo biloba leaf extract | Injection | 2017TanD | 57.6%(19/33) | Ginkgo biloba extract | 1(100.0%) |
|  |  | 2020JiHJ | 48.6%(17/35) |  |  |
| Multi-Ginkgo biloba Preparations | Mixed forms | 2006LiKJ | NA | Ginkgo biloba extract | 8(80.0%) |
|  |  | 2012MaLH | 34.1%(29/85) |  |  |
|  |  | 2015QinSC | 22.3%(25/112) |  |  |
|  |  | 2015WangL | 21.4%(24/112) |  |  |
|  |  | 2015XuJY | 10.7%(12/112) |  |  |
|  |  | 2017DongWS | 8.3%(10/120) |  |  |
|  |  | 2018WangYS | 12.1%(15/124) |  |  |
|  |  | 2020ChongPZ | 9.5%(13/137) |  |  |
|  |  | 2021ZhaoS | 9.0%(13/145) |  |  |
|  |  | 2022LiTT | 9.6%(14/146) |  |  |
|  |  | 2025HuYQ | 6.0%(9/150) |  |  |
