# Supplementary material for: Natural product extracts for ischemic stroke: a methodological evaluation and meta-epidemiological analysis
Source: Front Pharmacol. 2026 Jan 5;16:1730699. doi: 10.3389/fphar.2025.1730699 (PMC12813109; doi:10.3389/fphar.2025.1730699)
Supplement: Supplementary file 2 [file Table2.pdf]

Table S2 Impact of critical and non-critical AMSTAR-2 item deficiencies on overall methodological quality ratings

		Critically Low (N=68)	Low/ moderate (N=11)	OR	P
**Item1**	N	9 (13.2%)	0 (0%)	NA	0.994
	Y/PY	59 (86.8%)	11 (100%)		
**Item2**	N	63 (92.6%)	0 (0%)	NA	0.995
	Y/PY	5 (7.4%)	11 (100%)		
**Item3**	N	0 (0%)	0 (0%)	NA	NA
	Y/PY	68 (100%)	11 (100%)		
**Item4**	N	6 (8.8%)	0 (0%)	NA	0.992
	Y/PY	62 (91.2%)	11 (100%)		
**Item5**	N	35 (51.5%)	0 (0%)	NA	0.992
	Y/PY	33 (48.5%)	11 (100%)		
**Item6**	N	23 (33.8%)	1 (9.1%)	5.11 (0.62-42.40)	0.131
	Y/PY	45 (66.2%)	10 (90.9%)		
**Item7**	N	68 (100%)	7 (63.6%)	NA	0.992
	Y/PY	0 (0%)	4 (36.4%)		
**Item8**	N	27 (39.7%)	0 (0%)	NA	0.993
	Y/PY	41 (60.3%)	11 (100%)		
**Item9**	N	21 (30.9%)	0 (0%)	NA	0.994
	Y/PY	47 (69.1%)	11 (100%)		
**Item10**	N	68 (100%)	11 (100%)	NA	NA
	Y/PY	0 (0%)	0 (0%)		
**Item11**	N	42 (61.8%)	1 (9.1%)	16.15 (1.95-133.64)	0.010
	Y/PY	26 (38.2%)	10 (90.9%)		
**Item12**	N	66 (97.1%)	11 (100%)	NA	0.993
	Y/PY	2 (2.9%)	0 (0%)		
**Item13**	N	14 (20.6%)	0 (0%)	NA	0.992
	Y/PY	54 (79.4%)	11 (100%)		
**Item14**	N	50 (73.5%)	3 (27.3%)	7.41 (1.77-31.02)	0.006
	Y/PY	18 (26.5%)	8 (72.7%)		
**Item15**	N	17 (25%)	0 (0%)	NA	0.991
	Y/PY/NA	51 (75%)	11 (100%)		
**Item16**	N	44 (64.7%)	1 (9.1%)	18.33 (2.21-151.96)	0.007
	Y/PY	24 (35.3%)	10 (90.9%)		
