# Supplementary material for: Natural product extracts for ischemic stroke: a methodological evaluation and meta-epidemiological analysis
Source: Front Pharmacol. 2026 Jan 5;16:1730699. doi: 10.3389/fphar.2025.1730699 (PMC12813109; doi:10.3389/fphar.2025.1730699)
Supplement: Supplementary file 3 [file Supplementaryfile1.pdf]

Supplementary File 1

Search Strategy

Phase 1: Initial Systematic Search

A AND B AND C

**A**	systematic review[Title/Abstract] OR meta analysis[Title/Abstract])
**B**	ischemic stroke[Title/Abstract] OR cerebral infarction[Title/Abstract] OR cerebral ischemia[Title/Abstract] OR acute ischemic stroke[Title/Abstract]
**C**	(ginkgo[Title/Abstract] OR ginkgolide[Title/Abstract] OR "ginkgo ketone ester"[Title/Abstract] OR "ginkgo diterpene lactone*"[Title/Abstract] OR Shuxuening[Title/Abstract] OR danhong[Title/Abstract] OR Salvia[Title/Abstract] OR "Salvia miltiorrhiza"[Title/Abstract] OR safflower[Title/Abstract] OR "Carthamus tinctorius"[Title/Abstract] OR sanqi[Title/Abstract] OR "Panax notoginseng"[Title/Abstract] OR "Panax notoginseng saponins"[Title/Abstract] OR xuesaitong[Title/Abstract] OR "Ixeris sonchifolia"[Title/Abstract] OR kudiezi[Title/Abstract] OR dengzhan[Title/Abstract] OR Breviscapine[Title/Abstract] OR "Erigeron breviscapus"[Title/Abstract] OR Puerarin[Title/Abstract] OR "Pueraria lobata"[Title/Abstract] OR chuanxiong[Title/Abstract] OR chuanxiongqin[Title/Abstract] OR Tetramethylpyrazine[Title/Abstract] OR shenxiong[Title/Abstract] OR shenmai[Title/Abstract] OR shengmai[Title/Abstract] OR Gastrodin[Title/Abstract] OR shuxuetong[Title/Abstract] OR Coniferin[Title/Abstract] OR Quercetin[Title/Abstract] OR Epigallocatechin gallate[Title/Abstract] OR Resveratrol[Title/Abstract] OR Apocynin[Title/Abstract] OR Baicalin[Title/Abstract] OR Naringin[Title/Abstract] OR Triptolide[Title/Abstract] OR Phycocyanobilin[Title/Abstract])

Phase 2: Supplemental Search of Regional Databases

Database	Search strategy
CNKI	TKA=(‘系统综述’+‘系统评价’+‘meta’) AND TKA=(‘银杏’+‘金纳多’+‘三七总皂苷’+‘舒血宁’+‘血塞通’+‘三七’+‘血栓通’) AND TKA=(‘脑梗死’+‘脑梗’+‘腔隙性脑梗’+ ‘腔梗’+‘脑栓塞’+‘脑血栓’+‘脑卒中’+‘卒中’+‘中风’+‘脑缺血’+‘脑血管病’+ ‘缺血性脑中风’ +‘缺血性脑卒中’+‘缺血性中风’+‘缺血性卒中’)
VIP	(M=(系统综述 OR 系统评价 OR meta) OR R=(系统综述 OR 系统评价 OR meta)) AND (M=(银杏 OR 金纳多 OR 三七 OR 舒血宁 OR 血塞通 OR 血栓通) OR R=(银杏 OR 金纳多 OR 三七 OR 舒血宁 OR 血塞通 OR 血栓通)) AND (M=(脑梗 OR 腔梗 OR 脑栓塞 OR 脑血栓 OR 卒中 OR 中风 OR 脑缺血 OR 脑血管病) OR R=(脑梗 OR 腔梗 OR 脑栓塞 OR 脑血栓 OR 卒中 OR 中风 OR 脑缺血 OR 脑血管病))
WANFANG	主题:("系统综述" or "系统评价" or "meta") and 主题:("银杏" or "金纳多" or "三七" or "舒血宁" or "血塞通" or "血栓通") and 主题:("脑梗" or "腔梗" or "脑栓塞" or "脑血栓" or "卒中" or "中风" or "脑缺血" or "脑血管病")
KoreaMed	("systematic review"[TIAB] OR "meta analysis"[TIAB]) AND ("ischemic stroke"[TIAB] OR "cerebral infarction"[TIAB] OR "cerebral ischemia"[TIAB]) AND ("ginkgo"[TIAB] OR "ginkgolide"[TIAB] OR "ginkgo ketone ester"[TIAB] OR "ginkgo diterpene lactone"[TIAB] OR "Panax notoginseng"[TIAB])
KMbase	(((체계적 문헌고찰|title) OR (메타분석|title) OR (체계적综述|title) OR (체계적 문헌고찰|abstract) OR (메타분석|abstract)) AND ((허혈성

	뇌졸중|title) OR (뇌경색|title) OR (뇌허혈|title) OR (허혈성 뇌졸중|abstract) OR (뇌경색|abstract) OR (뇌허혈|abstract)) AND ((은행|title) OR (은행엽|title) OR (은행나무|title) OR (긍코라이드|title) OR (빌로발라이드|title) OR (삼칠|title) OR (파낙스 노토진센|title) OR (은행|abstract) OR (은행엽|abstract) OR (은행나무|abstract) OR (긍코라이드|abstract) OR (빌로발라이드|abstract) OR (삼칠|abstract) OR (파낙스 노토진센|abstract)))
**J-STAGE**	((ABST:("イチョウ" OR "ギンコウ" OR "銀杏") AND ABST:("抽出物" OR "エキス" OR "治療" OR "効果")) OR (ABST:("サンシチ" OR "田七" OR "三七") AND ABST:("抽出物" OR "エキス" OR "治療" OR "効果"))) AND ABST:("脳梗塞" OR "虚血性脳卒中") AND ABST:("システマティックレビュー" OR "系統的レビュー" OR "メタアナリシス")

Abbreviations: CNKI: China National Knowledge Infrastructure (https://www.cnki.net/); VIP: China Science and Technology Journal Database (http://www.cqvip.com/); WANFANG: Wanfang Data Knowledge Service Platform (http://www.wanfangdata.com.cn/); KoreaMed: Korean Medical Database (https://koreamed.org/); KMbase: Korean Medical Database (https://kmbase.medric.or.kr/); J-STAGE: Japan Science and Technology Information Aggregator, Electronic (https://www.jstage.jst.go.jp/)
