# Supplementary material for: Natural product extracts for ischemic stroke: a methodological evaluation and meta-epidemiological analysis
Source: Front Pharmacol. 2026 Jan 5;16:1730699. doi: 10.3389/fphar.2025.1730699 (PMC12813109; doi:10.3389/fphar.2025.1730699)
Supplement: Supplementary file 4 [file Supplementaryfile2.pdf]

Supplementary File 2

Records Screened at Title and Abstract with Exclusion Reasons

Phase 1: Initial Systematic Search

ID	Title	Reasons for exclusion
2007WuB	Meta-analysis of traditional Chinese patent medicine for ischemic stroke.	Interventions including non-NPEs
2012WangB	Meta-analysis of the clinical effect of ligustrazine on diabetic nephropathy.	Mixed Population
2014FanSY	Safflower yellow for acute ischemic stroke: A systematic review of randomized controlled trials.	include
2014YinB	Ginkgo biloba on focal cerebral ischemia: a systematic review and meta-analysis.	Preclinical Study
2015WangL	[System evaluation on Ginkgo Biloba extract in the treatment of acute cerebral infarction].	include
2015ZhangXM	Xuesaitong injection as one adjuvant treatment of acute cerebral infarction: a systematic review and meta-analysis.	include
2016LiuXT	Effectiveness and safety of ShenXiong glucose injection for acute ischemic stroke: a systematic review and GRADE approach.	Non-RCTs included
2016WangH	An Overview of Systematic Reviews of Danhong Injection for Ischemic Stroke.	overview of SRs
2017LiJG	Chinese herbal medicine Dengzhan Xixin injection for acute ischemic stroke: A systematic review and meta-analysis of randomised controlled trials.	include
2017WangKH	A comparative study of Danhong injection and Salvia miltiorrhiza injection in the treatment of cerebral infarction: A systematic review and meta-analysis.	include
2017ZhangYF	[Meta-analysis on indirect comparison of Erigeron breviscapus injection and Breviscapus injection in treatment of acute ischemic stroke].	include
2017ZhengQH	Efficacy and safety of puerarin injection in curing acute ischemic stroke: A meta-analysis of randomized controlled trials.	include

2018ChenZJ	A systematic review on the rhizome of Ligusticum chuanxiong Hort. (Chuanxiong).	Preclinical Study
2018ChledzikS	Pharmacological Effects of Scutellarin, An Active Component of Genus Scutellaria and Erigeron: A Systematic Review.	Preclinical Study
2018LiuS	Comparative efficacy of Chinese herbal injections for treating acute cerebral infarction: a network meta-analysis of randomized controlled trials.	Network Meta-analysis
2018TanD	Injections of ginkgo in the treatment of cerebral infarction: a systematic review and network Meta-analysis.	Network Meta-analysis
2018XieBC	[Systematic review on efficacy and safety of Danshen Chuanxiongqin Injection in treatment of acute cerebral infarction].	include
2018XieBC	Efficacy and Safety of the Injection of the Traditional Chinese Medicine Puerarin for the Treatment of Diabetic Peripheral Neuropathy: A Systematic Review and Meta-Analysis of 53 Randomized Controlled Trials.	Mixed Population
2018YangXY	Chinese herbal medicine Dengzhan Shengmai capsule as adjunctive treatment for ischemic stroke: A systematic review and meta-analysis of randomized clinical trials.	Interventions including non-NPEs
2019DuanXJ	Comparative study of xuesaitong injection and compound salvia miltiorrhizae injection in the treatment of acute cerebral infarction: a meta-analysis.	include
2019JinLR	Effects of six types of aspirin combination medications for treatment of acute cerebral infarction in China: A network meta-analysis.	Network Meta-analysis
2019LiuS	Efficacy of Danshen Class Injection in the Treatment of Acute Cerebral Infarction: A Bayesian Network Meta-Analysis of Randomized Controlled Trials.	Network Meta-analysis
2019LiuYY	Ginkgo leaf extract and dipyridamole injection as adjuvant treatment for acute cerebral infarction: Protocol for systemic review and meta-analysis of randomized controlled trials.	protocol
2019WuLH	The Effects of Breviscapine Injection on Hypertension in Hypertension-Induced Renal Damage Patients: A Systematic Review and a Meta-Analysis.	Mixed Population

2019XueP	Efficacy and Safety of Ginkgo Leaf Extract and Dipyridamole Injection for Ischemic Stroke: A Systematic Review and Meta Analysis.	include
2019YuDD	[Systematic review and Meta-analysis on randomized controlled trials on effectiveness and safety of Kudiezi Injection in treatment of acute cerebral infarction].	include
2020ChongPZ	Efficacy and Safety of Ginkgo biloba in Patients with Acute Ischemic Stroke: A Systematic Review and Meta-Analysis.	include
2020FangH	Effects of shuxuetong injection for cerebral infarction: A protocol for systematic review and meta-analysis.	protocol
2020JiHJ	Ginkgol Biloba extract as an adjunctive treatment for ischemic stroke: A systematic review and meta-analysis of randomized clinical trials.	include
2020LyuJ	Clinical evidence and GRADE assessment for breviscapine injection (DengZhanHuaSu) in patients with acute cerebral infarction.	include
2020SunT	Effect of Panax notoginseng Saponins on Focal Cerebral Ischemia-Reperfusion in Rat Models: A Meta-Analysis.	Preclinical Study
2020YangF	Panax notoginseng for Cerebral Ischemia: A Systematic Review.	Preclinical Study
2020ZhouDR	Clinical Efficacy of Tonic Traditional Chinese Medicine Injection on Acute Cerebral Infarction: A Bayesian Network Meta-Analysis.	Network Meta-analysis
2021FengL	The efficacy and safety of Xuesaitong injection combined with western medicines in the treatment of ischemic stroke: an updated systematic review and meta-analysis.	include
2021GaoY	[Overview of systematic reviews on Shuxuening Injection].	overview of SRs
2021GuanYJ	[Systematic review of efficacy and safety of Ginkgo biloba extract 50 in treatment of ischemic stroke].	include
2021HuRX	[Exploring efficacy of Chinese medicine injection for promoting blood circulation and removing blood stasis in treatment of acute cerebral infarction based on two complex network analysis methods].	Network Meta-analysis

2021LiJL	Comparison of Traditional Chinese Medicine in the Long-Term Secondary Prevention for Patients with Ischemic Stroke: A Systematical Analysis.	Non-Functional outcomes reported
2021LiuJ	Resveratrol has an Overall Neuroprotective Role in Ischemic Stroke: A Meta-Analysis in Rodents	Preclinical Study
2021LiY	Safflower yellow pigment and Sanqi Panax notoginseng in the treatment of acute cerebral infarction: a systematic review, meta-analysis, and cost-effectiveness analysis.	include
2021MengTT	[Systematic review and Meta-analysis of clinical efficacy and safety of Ginkgo Leaf Tablets in treatment of acute cerebral infarction].	include
2021WangLD	Systematic Review and Meta-Analysis on Randomized Controlled Trials on Efficacy and Safety of Panax Notoginseng Saponins in Treatment of Acute Ischemic Stroke.	include
2021ZhaoS	The Clinical Efficacy of Ginkgo biloba Leaf Preparation on Ischemic Stroke: A Systematic Review and Meta-Analysis.	include
2022DaiLL	Panax notoginseng preparation plus aspirin versus aspirin alone on platelet aggregation and coagulation in patients with coronary heart disease or ischemic stroke: A meta-analysis of randomized controlled trials.	Mixed Population
2022GaoYJ	The efficacy and safety of the Xuesaitong soft capsule in the treatment of patients with ischemic stroke: systematic review and meta-analysis.	include
2022GengHJ	Xuesaitong oral preparation as adjuvant therapy for treating acute cerebral infarction: A systematic review and meta-analysis of randomized controlled trials.	include
2022GuoC	Effect and Mechanisms of Quercetin for Experimental Focal Cerebral Ischemia: A Systematic Review and Meta-Analysis	Preclinical Study
2022LiL	Comparative efficacy of Honghua class injections for treating acute ischemic stroke: A Bayesian network meta-analysis of randomized controlled trials.	Network Meta-analysis
2022LiTT	Platelet-activating factor receptor antagonists of natural origin for acute ischemic stroke: a systematic review of current evidence.	include
2022LiuFM	[Clinical comprehensive evaluation of Ginkgolide Injection in treatment of cerebral infarction]	non-SRs

2022LiuXY	Updated evidence of Dengzhan Shengmai capsule against ischemic stroke: A systematic review and meta-analysis.	Interventions including non-NPEs
2022LiYB	The Efficacy and Safety of Ischemic Stroke Therapies: An Umbrella Review.	umbrella review
2022MaY	The add-on effects of Danhong injection among patients with ischemic stroke receiving Western medicines: A systematic review and meta-analysis.	include
2022XueR	A meta-analysis of resveratrol protects against cerebral ischemia/reperfusion injury: Evidence from rats studies and insight into molecular mechanisms	Preclinical Study
2022ZhaoH	The Efficacy and Safety of Ginkgo Terpene Lactone Preparations in the Treatment of Ischemic Stroke: A Systematic Review and Meta-Analysis of Randomized Clinical Trials.	include
2023LiLD	Edaravone combined with Shuxuening versus edaravone alone in the treatment of acute cerebral infarction: A systematic review and meta-analysis.	include
2023López-MoralesMA	Effect and mechanisms of resveratrol in animal models of ischemic stroke: A systematic review and Bayesian meta-analysis	Preclinical Study
2023PengSX	[Network Meta-analysis of Chinese medicine injections for activating blood and resolving stasis in adjuvant treatment of acute ischemic stroke].	Network Meta-analysis
2023ShiXY	Efficacy and safety of Panax notoginseng saponins (Xuesaitong) for patients with acute ischemic stroke: a systematic review and meta-analysis of randomized controlled trials.	include
2023WangQY	Chemical composition, pharmacology and pharmacokinetic studies of GuHong injection in the treatment of ischemic stroke.	Preclinical Study
2023XieXZ	The protective effect of an extract of Salvia miltiorrhiza Bunge (Danshen) on cerebral ischemic injury in animal models: A systematic review and meta-analysis.	Preclinical Study
2023YanMY	Ginkgolide injections in meglumine, combined with edaravone, significantly increases the efficacy in acute ischemic stroke: A meta-analysis.	include

2023ZhuLJ	[Network Meta-analysis of Qi-benefiting and blood-activating Chinese patent medicines against ischemic stroke].	Network Meta-analysis
2024LiM	Efficacy analysis of neuroprotective drugs in patients with acute ischemic stroke based on network meta-analysis.	Network Meta-analysis
2024LiuYY	The use of Panax notoginseng saponins injections after intravenous thrombolysis in acute ischemic stroke: a systematic review and meta-analysis.	include
2024LyuYT	Efficacy and safety of edaravone combined with Ginkgo Leaf Extract and Dipyridamole in the treatment of acute cerebral infarction: A systematic review and meta-analysis.	Non-RCTs included
2024MaZY	Safety and effectiveness of Salvia miltiorrhiza and ligustrazine injection for acute cerebral infarction in Chinese population: a PRISMA-compliant meta-analysis.	include
2024MengTT	Chinese herbal medicine Ginkgo biloba L. preparations for ischemic stroke: An overview of systematic reviews and meta-analyses.	overview of SRs
2024WangJ	Exploring the therapeutic efficacy and pharmacological mechanism of Guizhi Fuling Pill on ischemic stroke: a meta-analysis and network pharmacology analysis	Preclinical Study
2024WangJS	Role of salvianolic acid B in the treatment of acute ischemic stroke: a systematic review and meta-analysis of animal models.	Preclinical Study
2024XinSL	Meta-analysis of the efficacy and safety of Ginkgolide Meglumine Injection combined with Butylphthalide in the treatment of Acute Ischemic Stroke.	Non-RCTs included
2024YangF	Therapeutic efficacy and pharmacological mechanism of Yindan Xinnaotong soft capsule on acute ischemic stroke: a meta-analysis and network pharmacology analysis	Preclinical Study
2024YiYH	Comparative efficacy and safety of traditional Chinese medicine injections in patients with transient ischemic attack: A systematic review and network meta-analysis.	Network Meta-analysis
2024ZhanJ	Shuxuening injection for treating acute ischemic stroke: a PRISMA-compliant systematic review and meta-analysis of randomized controlled trials.	include

2024ZhaoA	Substance basis and pharmacological mechanism of heat-clearing herbs in the treatment of ischaemic encephalopathy: a systematic review and network pharmacology	Preclinical Study
2024ZhaoS	Efficacy and safety of Shenmai injection for acute ischemic stroke: a systematic review and meta-analysis.	Interventions including non-NPEs
2025LuoX	The potential value of traditional Chinese medicine monomers in cerebral ischemia-reperfusion injury: a network meta-analysis based on animal model.	Network Meta-analysis
2025WangLD	Ginkgo diterpene lactone meglumine for functional recovery in patients with acute ischemic stroke: A systematic review and meta-analysis.	include
2025XuH	The efficacy and safety of ginkgo terpene lactone preparations combined with antiplatelet aents in the treatment of ischemic stroke: a systematic review and meta-analysis.	include

Phase 2: Supplemental Search of Regional Databases

ID	Title	Reasons for exclusion
2007LiKJ	缺血性卒中急性期中医优势治法方药研究	Duplicate Publication
2008LiKJ	缺血性卒中“病机-证候-治法-方药”体系构建	Duplicate Publication
2008MengLJ	缺血性卒中病机假说——“血瘀生风”临床依据研究	Duplicate Publication
2016LiKJ	病机-证治体系规范化模式构建及其在两种重大疾病中的应用	Duplicate Publication
2010LiHJ	三七治疗急性缺血性卒中	Full text unavailable
2011ZhongJM	银杏叶提取物治疗急性脑梗死随机对照试验的 Meta 分析	Full text unavailable
2014ZhangL	奥扎格雷钠联合舒血宁注射液治疗急性脑梗死的系统评价	Full text unavailable
2016MaL	血塞通注射液治疗脑梗死的 Meta 分析	Full text unavailable
2009DuXX	疏血通治疗急性脑梗死疗效的 Meta 分析	Full text unavailable
2011NiJN	银杏达莫注射液临床应用的系统评价	Full text unavailable
2013GuSY	奥扎格雷与舒血宁治疗急性脑梗死的 Meta 分析	Full text unavailable
2014LiangY	舒血宁治疗急性脑血管疾病的疗效及安全性评价 JF	Full text unavailable
2019GengHJ	血塞通口服制剂治疗缺血性中风病有效性系统评价与 Meta 分析	Full text unavailable
2006LiKJ	银杏叶制剂治疗缺血性中风急性期随机对照试验的系统评价	include
2007LiKJ	三七制剂治疗缺血性中风急性期随机对照试验的系统评价	include
2008NiSQ	银杏达莫注射液治疗急性脑梗死随机对照试验的 Meta 分析	include
2009ChenB	三七总皂甙注射液对急性期脑梗死治疗的系统评价	include
2009DingX	血塞通注射液治疗急性脑梗死的 Meta 分析	include
2012LiHT	血栓通辅助治疗急性脑梗死有效性和安全性的 Meta 分析	include
2012MaLH	银杏叶制剂治疗缺血性中风急性期临床疗效的系统评价	include
2012TianCJ	血塞通注射液治疗缺血性中风急性期临床疗效的系统评价	include
2012XiBC	舒血宁注射液治疗急性脑梗死病人的荟萃分析	include
2013ChenJ	血栓通治疗急性脑梗死的 Meta 分析	include
2013XiaW	三七通舒治疗缺血性脑卒中临床疗效及安全性系统评价	include

2013YuZW	银杏达莫注射液联合巴曲酶治疗脑梗死疗效的 Meta 分析	include
2014HuJH	血宁治疗急性脑梗死的 Meta 分析	include
2014LiT	依达拉奉联合舒血宁治疗脑梗死的系统评价	include
2014ZhangY	血塞通注射液治疗急性脑梗塞的疗效与安全性系统评价	include
2014ZhengCJ	奥扎格雷钠联合血栓通注射液治疗急性脑梗死疗效的系统评价	include
2015QinSC	银杏叶提取物治疗急性脑梗死随机对照试验的 Meta 分析	include
2015WangL	银杏叶提取物治疗急性脑梗死的系统评价	include
2015XuJY	银杏叶注射液治疗急性脑梗死随机对照试验的系统评价	include
2016QiJ	三七制剂治疗急性中风临床疗效的系统评价	include
2016RenDQ	银杏达莫注射液治疗急性脑梗死的 Meta 分析	include
2016WangQ	基于 Meta 分析的血栓通注射剂治疗急性脑梗死临床评价研究	include
2017DongWS	银杏叶提取物与三七总皂苷辅助治疗急性脑梗死临床疗效及安全性的 Meta 分析	include
2017TanD	基于 Meta 分析的银杏叶提取物注射液治疗脑梗死临床评价研究	include
2017TianP	血塞通治疗急性缺血性脑卒中随机对照试验的系统评价	include
2017WuFB	丁苯酞胶囊联合血栓通注射液治疗急性脑梗死疗效和安全性的 Meta 分析	include
2017ZhaoMR	纤溶酶联合血栓通注射液治疗急性缺血性脑卒中疗效和安全性的 Meta 分析	include
2018ChenX	三七通舒胶囊治疗急性缺血性脑卒中的临床疗效 Meta 分析	include
2018ChengMZ	血栓通联合依达拉奉治疗急性脑梗死的 Meta 分析	include
2018WangQ	银杏二萜内酯葡胺注射液治疗急性脑梗死临床疗效系统评价	include
2018WangYS	脑卒中银杏注射液疗效的系统评价、早期风险因素评估和预防策略	include
2019JinFH	银杏二萜内酯葡胺注射液治疗脑梗死的疗效及安全性的 Meta 分析	include
2019LiuNN	血塞通注射液联合奥扎格雷钠治疗急性脑梗死有效性与安全性的 Meta 分析	include
2020SunXY	联合血塞通对比单纯西药治疗脑梗塞临床疗效的 Meta 分析	include
2021GuanYJ	银杏酮酯治疗缺血性脑卒中疗效及安全性的系统评价	include

2021LuoH	银杏内酯注射液联合阿替普酶治疗急性缺血性卒中的 Meta 分析	include
2021MengTT	银杏内酯注射液辅助治疗急性缺血性脑卒中的系统评价与 Meta 分析	include
2021MengTT	银杏叶片治疗急性脑梗死临床疗效和安全性的系统评价与 Meta 分析	include
2021RenFQ	血栓通注射液改善急性脑梗死患者炎症因子水平的 Meta 分析	include
2021YanMY	血塞通联合依达拉奉对急性脑梗死患者血液流变学影响的 meta 分析	include
2021ZhangLL	银杏二萜内酯葡胺注射液联合西药治疗脑梗死的疗效和安全性 Meta 分析	include
2022FengCN	血塞通软胶囊治疗脑梗死有效性和安全性的系统评价	include
2022LiJQ	血塞通胶囊治疗脑梗死的临床疗效与血液流变学 Meta 分析	include
2023FengH	血栓通注射剂治疗急性脑梗死有效性及抗凝血指标的 meta 分析	include
2023SongGL	注射用血栓通（冻干）治疗心脑血管疾病有效性和安全性的临床证据综合评价	include
2023WangYT	探讨血塞通在增强阿托伐他汀治疗糖尿病合并脑梗死方面的疗效及作用机制	include
2024ChenZW	银杏叶片治疗缺血性中风的 Meta 分析	include
2024LiXK	基于真实世界的血塞通软胶囊对脑梗死患者血液流变学及血脂的影响	include
2025HuYQ	静脉应用银杏叶制剂治疗急性缺血性卒中有效性和安全性的 meta 分析	include
2022ZhengT	活血类中成药治疗卒中组方规律及脑心通联合丁苯酞治疗卒中 Meta 分析	Interventions including non- GBEs/PNEs
2001GuoJW	活血化瘀中药及复方治疗急性脑出血的 Meta 分析	Interventions including non- GBEs/PNEs
2005MaLH	系统评价活血化瘀法治疗缺血性中风急性期的临床疗效研究	Interventions including non- GBEs/PNEs
2005WuB	中成药治疗缺血性脑卒中的优势药物研究	Interventions including non- GBEs/PNEs
2006LiKJ	不同水蛭制剂治疗急性期缺血性脑卒中临床疗效比较	Interventions including non- GBEs/PNEs
2006WangJS	三七总皂苷和复方丹参注射液治疗脑梗死的 Meta 分析	Interventions including non- GBEs/PNEs
2010HuangH	前、后循环超急性期脑梗死动脉溶栓治疗的临床分析	Interventions including non- GBEs/PNEs
2010YiZM	马来酸桂哌齐特治疗急性脑梗塞的系统评价	Interventions including non- GBEs/PNEs

2011CuiHJ	红花黄色素注射液治疗急性脑梗死总有效率和安全性的 Meta 分析	Interventions including non- GBEs/PNEs
2011LiY	银杏达莫与丹参注射剂对照急性脑梗死的 Meta 分析	Interventions including non- GBEs/PNEs
2012LiuXH	高压氧治疗急性椎基底动脉系统脑梗死疗效观察	Interventions including non- GBEs/PNEs
2012MaAX	我国依达拉奉联合奥扎格雷与单用奥扎格雷治疗缺血性脑卒中的疗效比较——基于 NDS 有效率、NDS 评分的 Meta 分析	Interventions including non- GBEs/PNEs
2013LiuJ	苦碟子注射液治疗急性脑梗死适应证候的研究	Interventions including non- GBEs/PNEs
2013MaAX	我国依达拉奉联合奥扎格雷与单用奥扎格雷治疗急性缺血性脑卒中疗效比较的 Meta 分析	Interventions including non- GBEs/PNEs
2013WangHT	疏血通注射剂治疗脑梗死的用药有效性、安全性及经济性评价	Interventions including non- GBEs/PNEs
2013WuGD	球囊扩张支架成形术治疗症状性 MCA 狭窄的临床观察	Interventions including non- GBEs/PNEs
2014GengYT	舒脑欣滴丸佐治后循环缺血性眩晕血瘀证临床研究	Interventions including non- GBEs/PNEs
2014LanXW	血塞通注射液与复方丹参注射液比较治疗急性脑梗死的系统评价	Interventions including non- GBEs/PNEs
2014MaLH	缺血性中风血瘀生风病机假说的循证检验研究	Interventions including non- GBEs/PNEs
2014QiYJ	补阳还五汤加减联合血塞通注射液治疗脑梗塞的系统评价	Interventions including non- GBEs/PNEs
2015MaWW	疏血通与血塞通治疗急性脑梗死疗效比较的 meta 分析	Interventions including non- GBEs/PNEs
2015XieHJ	阿司匹林联合活血化瘀类中成药治疗老年缺血性脑卒中效果与安全性的 Meta 分析	Interventions including non- GBEs/PNEs
2017LiJ	疏血通注射液治疗急性脑梗死临床疗效和安全性的系统评价	Interventions including non- GBEs/PNEs
2017LiS	红花黄色素注射液治疗脑梗死疗效及安全性的系统评价	Interventions including non- GBEs/PNEs
2017LiuM	糖尿病性脑血管病的循证病机—证治体系构建	Interventions including non- GBEs/PNEs
2017ZhangD	基于 Meta 分析的红花注射剂治疗急性脑梗死临床评价研究	Interventions including non- GBEs/PNEs
2018GaoF	瓜蒌皮注射液治疗急性脑梗死的疗效和安全性 Meta 分析	Interventions including non- GBEs/PNEs
2019AnJP	系统评价补阳还五汤加减联合血塞通治疗脑梗塞的临床疗效	Interventions including non- GBEs/PNEs
2019XuMB	中药对急性脑梗死临床疗效及安全性的系统评价及 Meta 分析	Interventions including non- GBEs/PNEs

2021HanXC	分析血栓通联合丹参多酚盐对急性缺血性脑卒中患者认知障碍及凝血-纤溶系统影响的临床研究	Interventions including non- GBEs/PNEs
2021TangJP	注射用丹参多酚酸治疗缺血性脑卒中的临床疗效和代谢组学研究	Interventions including non- GBEs/PNEs
2022LiXJ	补阳还五汤联合血塞通注射液治疗脑梗塞的系统评价	Interventions including non- GBEs/PNEs
2016ChenHJ	疏血通与三七总皂苷治疗急性脑梗死临床疗效及安全性比较的 Meta 分析	Interventions including non- GBEs/PNEs
2020WeiBN	奥扎格雷钠联合银杏达莫治疗急性脑梗死有效性和安全性的 Meta 分析	Interventions including non- GBEs/PNEs
2012PengXQ	依达拉奉联合银杏达莫治疗急性脑梗死的 Meta 分析	Interventions including non- GBEs/PNEs
2014ChaiJ	奥扎格雷钠联合舒血宁注射液治疗急性脑梗死的系统评价	Interventions including non- GBEs/PNEs
2015ZouC	血塞通注射液联合依达拉奉治疗急性脑梗死的 Meta 分析	Interventions including non- GBEs/PNEs
2017LiuJY	舒血宁注射液单用与联合奥扎格雷钠治疗急性脑梗死疗效比较的 Meta 分析	Interventions including non- GBEs/PNEs
2021WangJC	奥扎格雷钠联合红花注射液治疗急性脑梗死的 Meta 分析	Interventions including non- GBEs/PNEs
2017ZhangYD	丹红与血塞通治疗脑梗死的药物经济学评价	Interventions including non- GBEs/PNEs
2012ShiR	血栓通注射液治疗缺血性中风急性期临床疗效的系统评价	Interventions including non- GBEs/PNEs
2013ZhangSS	基于系统评价比较三七制剂在不同类型中风中的疗效优势	Interventions including non- GBEs/PNEs
2013MaLH	口服三七制剂治疗缺血性中风急性期临床疗效的系统评价	Interventions including non- GBEs/PNEs
2009YuZS	三七总皂苷和三七复方治疗脑出血的系统评价	Mixed Population
2011DongQ	中药舒血宁注射液治疗短暂性脑缺血发作有效性的系统评价	Mixed Population
2012MaLH	血塞通注射液治疗出血性中风急性期临床疗效的系统评价	Mixed Population
2013ZhangSS	银杏叶制剂治疗出血性中风急性期随机对照试验的系统评价研究	Mixed Population
2014MaLH	出血性中风血瘀生风病机假说的循证检验研究	Mixed Population
2015WangR	基于指南和临床路径的椎-基底动脉供血不足住院患者用药分析与评价	Mixed Population
2015XiaKZ	银杏达莫联合阿司匹林治疗短暂性脑缺血发作的疗效观察	Mixed Population

2015ZhangXM	基于 Meta 分析的中药注射剂治疗心脑血管疾病系统评价研究	Mixed Population
2017XiaoXL	非瓣膜性房颤的危险因素分析	Mixed Population
2019ChaiLL	银杏叶制剂治疗高脂血症的疗效及作用机制研究	Mixed Population
2020MaL	银杏叶片治疗血瘀证的 Meta 分析和功效成分研究	mixed population
2022LiuTC	新型口服抗凝剂和传统抗凝剂在需要抗凝治疗的肝硬化患者中的疗效与安全性比较	Mixed Population
2022ZhaoS	银杏酮酯滴丸治疗慢性脑供血不足的 Meta 分析及临床研究	Mixed Population
2023DaiLL	冠心病血瘀证血小板差异 miRNA 的临床验证及三七总皂苷基于 miR-223-3p 抗血小板作用机制研究	Mixed Population
2023LinJH	基于回顾性研究探讨中药治疗肝肾阴虚型老年高血压疗效	Mixed Population
2023WangY	降压中成药联合西药治疗高血压病的证据评价及循证实践研究	Mixed Population
2024SongWS	银杏叶提取物注射液辅助治疗脑出血疗效和安全性的 Meta 分析	Mixed Population
2024WangXY	基于真实世界数据冠脉临界病变中西医结合治疗的临床评价研究	Mixed Population
2017XiangY	活血化瘀类中药注射剂治疗中风的效果比较研究	Network Meta-analysis
2018TanD	基于贝叶斯网状 Meta 分析的银杏类注射剂临床评价研究	Network Meta-analysis
2020CuiRZ	口服中成药治疗急性脑梗死的网状 Meta 分析	Network Meta-analysis
2020LiJ	9 种活血化瘀类中药注射剂治疗缺血性脑卒中的网状 Meta 分析	Network Meta-analysis
2020YuDD	基于复杂网络方法评价活血化瘀中药注射剂治疗急性脑梗死的疗效	Network Meta-analysis
2021XieXL	三七类口服制剂治疗急性脑梗死的网状 Meta 分析	Network Meta-analysis
2022JingJK	3 种药物治疗急性缺血性脑卒中的网状 Meta 分析和药物经济学评价	Network Meta-analysis
2022RenP	中药注射液治疗高血压病脑出血的网状 Meta 分析	Network Meta-analysis
2022ZhengYH	活血化瘀类口服中成药治疗急性缺血性脑卒中有效性的网状 meta 分析	Network Meta-analysis
2023BianSB	中药注射液治疗急性脑出血有效性及安全性的网状 Meta 分析	Network Meta-analysis
2023GuanLY	6 种中药注射液治疗急性缺血性卒中的网状 meta 分析	Network Meta-analysis

2023LiuHX	5 种活血类中药注射剂联合化学药治疗急性缺血性脑卒中的贝叶斯网状 Meta 分析	Network Meta-analysis
2023PengSX	活血化瘀类中药注射液辅助治疗急性缺血性卒中的网状 Meta 分析	Network Meta-analysis
2023PengSX	十种活血类中药注射液辅助治疗急性缺血性卒中的网状 meta 分析	Network Meta-analysis
2023WangN	口服中成药联用西药治疗脑卒中后认知障碍的网状 Meta 分析	Network Meta-analysis
2024LinY	中药注射液联合丁苯酞治疗脑梗死临床疗效的网状 Meta 分析	Network Meta-analysis
2024PengT	6 种中药注射液治疗卒中后抑郁的网状 Meta 分析	Network Meta-analysis
2024SongYB	中成药治疗脑出血术后疗效的贝叶斯网状 Meta 分析	Network Meta-analysis
2025DuQL	中药注射液治疗脑小血管病的网状 Meta 分析	Network Meta-analysis
2025GongL	中药注射液联合常规药物治疗卒中后认知障碍的网状 Meta 分析	Network Meta-analysis
2025HanJ	不同中药注射液治疗短暂性脑缺血发作疗效的网状 Meta 分析	Network Meta-analysis
2025LinY	中药注射液联合依达拉奉治疗老年脑梗死的网状 Meta 分析	Network Meta-analysis
2025ZhuSJ	不同中成药注射液治疗后循环缺血性眩晕效果的网状 Meta 分析	Network Meta-analysis
2012ChenHY	血塞通注射液不良反应的 Meta 分析	non-functional outcome
2005FangXL	血塞通注射液协同爱通立对急性脑梗死静脉溶栓治疗的初步临床研究	non-RCTs included
2007ChenXY	三七治疗急性缺血性脑卒中疗效及安全性的系统评价	non-RCTs included
2008LiJ	金纳多注射液治疗急性缺血性脑卒中随机对照研究系统评价	non-RCTs included
2011HouJT	金纳多治疗脑梗死的系统评价	non-RCTs included
2011LiuJ	两种活血化瘀中药注射剂治疗急性缺血性脑卒中的风险评价及特点分析研究	non-RCTs included
2011YanJ	舒血宁注射液治疗脑梗死疗效的 Meta 分析	non-RCTs included
2012ZhengWK	舒血宁注射液治疗急性脑梗死的系统评价	non-RCTs included
2014LiuC	银杏叶提取物注射液治疗老年缺血性脑血管病的疗效与安全性的系统评价	non-RCTs included
2015ChenZP	依达拉奉联合血栓通治疗急性脑梗死临床研究 Meta 分析	non-RCTs included
2015LiLQ	依达拉奉联合血栓通治疗急性脑梗死临床研究的 Meta 分析	non-RCTs included
2018ZhaoH	银杏萜内酯类注射液治疗缺血性脑卒中疗效及安全性的系统评价	non-RCTs included

2010WeiXC	血塞通注射液治疗脑梗死疗效 Meta 分析	non-RCTs included
2008YangZG	中药三七对血液系统和心脑血管系统的影响	Non-SRs
2020HeZF	基于成本-效果和安全性评价的疏血通、舒血宁和丹红治疗急性脑梗死的随机对照研究	Non-SRs
2020LiC	奥扎格雷钠注射液治疗急性血栓性脑梗死的快速卫生技术评估	Non-SRs
2021GaoY	舒血宁注射液的系统评价再评价	Non-SRs
2021RenXL	银杏叶提取物注射液治疗缺血性脑卒中的快速卫生技术评估	Non-SRs
2021WangYL	基于 Meta 分析的血栓通联合天麻素注射液治疗脑血管疾病的药物经济学研究	Non-SRs
2022ZengC	奥扎格雷钠治疗脑梗死有效性、安全性及经济性的卫生技术评估	Non-SRs
2022LiuFM	银杏内酯注射液治疗脑梗死的临床综合评价	Non-SRs
2022ZhangBH	舒血宁注射液辅助治疗缺血性脑卒中的快速卫生技术评估	Non-SRs
2023ZhangBH	血塞通注射液治疗中风的快速卫生技术评估	Non-SRs
2023ZhangXM	注射用血栓通(冻干)治疗急性脑梗死的临床综合评价	Non-SRs
2007HuP	银杏达莫注射液治疗急性脑梗死有效性的 Meta 分析	Non-SRs
2012CaiLM	舒血宁注射液治疗急性脑梗死疗效的汇总分析	Non-SRs
2012GuoW	多指标系统评价血塞通联合银杏叶制剂治疗缺血性脑卒中的临床疗效观察	Non-SRs
2012LinY	血塞通注射液治疗脑梗死疗效 Meta 分析	Non-SRs
2014QinSZ	依达拉奉联合血栓通治疗急性脑梗死临床研究 meta 分析	Non-SRs
2014YuanX	丹红与血塞通治疗脑梗死的药物经济学评价	Non-SRs
2016JinYJ	奥扎格雷钠联合血栓通注射液治疗急性脑梗死疗效的系统评价	Non-SRs
2018WeiDX	云南民族药名方“桑芪首乌方”的成药性评价	Non-SRs
2018ZhangMY	血塞通注射液治疗中风的证据及评价	Non-SRs
2019LiGZ	血栓通注射液治疗心脑血管疾病系统评价的再评价	Non-SRs
2019ZhangD	血栓通注射液临床应用研究进展	Non-SRs
2020DongWS	基于循证医学方法评价四类不同有效成分中药注射剂治疗急性脑梗死疗效和安全性研究	Non-SRs
2021WangLD	三七总皂苷治疗急性脑梗死的系统评价再评价	Non-SRs
2022HuRX	中西医诊疗急性脑梗死超网络特性及诊疗要素的差异分析	Non-SRs

2022QiuWR	基于混合方法的血塞通软胶囊干预脑梗死的临床应用特征与共识研究	Non-SRs
2019WangSY	基于系统评价的银杏叶片治疗高血压病、脑梗死、冠心病心绞痛药物经济学分析	Non-SRs
2019WangY	丹红注射液和银杏达莫注射液治疗心脑血管疾病的系统评价及药物经济学对比分析	Non-SRs
2022ZhengH	基于系统评价的银杏达莫注射液治疗脑梗死的有效性与经济性评价	Non-SRs
2010HuangXP	黄芪总苷和三七总皂苷配伍对脑缺血再灌注后 MMP-9 和 TIMP-1 表达的影响	Preclinical Study
2010TangYH	三七总皂苷对脑缺血再灌注后 MMP-9 和 TIMP-1 表达的影响	Preclinical Study
2010YinCQ	三七对血液、心脑血管和中枢神经系统的药理作用研究概述	Preclinical Study
2010ZhouJC	银杏叶提取物对心血管系统的作用	Preclinical Study
2011ChenJM	三七总皂苷对心脑血管作用的药理研究新进展	Preclinical Study
2011ZhangX	丹参、三七有效组分及其复方的新剂型和制剂新技术研究进展	Preclinical Study
2013FuY	银杏叶提取物对中枢神经系统保护作用的研究进展	Preclinical Study
2013YangT	天麻素治疗神经系统疾病机制研究进展	Preclinical Study
2015QiuJ	银杏叶提取物对脑组织及脑神经细胞活性的保护机制研究	Preclinical Study
2017HuangWL	银杏内酯 A 对中枢神经系统作用及机制的研究进展	Preclinical Study
2017ShiYW	加味开心散颗粒的药学及其抗痴呆作用机理研究	Preclinical Study
2017WangT	脑血疏口服液与大骨瓣减压硬膜扩大减张缝合术治疗大面积脑梗死的疗效及对血清 NSE 和 hs-CRP 水平的影响	Preclinical Study
2020SunXY	血塞通上调小鼠脑微血管内皮细胞缺氧模型 VEGF 通路表达的作用机制	Preclinical Study
2020ZhuL	芪参益气方及其益气、活血组分对高血压大鼠血管舒张功能的调节机制探究	Preclinical Study
2021XingWJ	中药对脑缺血再灌注后血脑屏障的作用机制研究进展	Preclinical Study
2021MaZ	基于 ERK1/2 与 TGF-β1/smad3 通路研究参连复脉颗粒干预高血压心房重构及房颤易感性机制	Preclinical Study
2023BiL	关系视角下的脑缺血中水蛭-地龙方“咸寒”药性认识	Preclinical Study
2024LiuY	银杏内酯 B 口服递药系统的构建与评价研究	Preclinical Study
