# Supplementary material for: Natural product extracts for ischemic stroke: a methodological evaluation and meta-epidemiological analysis
Source: Front Pharmacol. 2026 Jan 5;16:1730699. doi: 10.3389/fphar.2025.1730699 (PMC12813109; doi:10.3389/fphar.2025.1730699)
Supplement: Supplementary file 5 [file Supplementaryfile3.pdf]

Supplementary File 3 Detailed redundancy classification process with PICO elements and individual reviewer ratings

ID	Populatiuon		Sample Size	Intervention			Comparison	Outcomes	Citation of previous studies	Redundancy Categories			Language
	Time of onset	Additional Inclusion Criteria		NPEs	Others	Duration				WSJ	PX	Final	
2007LiKJ	30d	/	1811	Multi-Panax notoginseng Preparations	CT	/	CT	Overall Effectiveness Rate; NIHSS	No	benchmark	benchmark	benchmark	Chinese
2016QiJ	/	TCM Syndrome	3060	Multi-Panax notoginseng Preparations	CT	/	placebo,CT	Overall Effectiveness Rate; NIHSS; BI score	No	Conceptual replication	Excessive replication	Conceptual replication	Chinese
2009ChenB	30d	/	1552	Multi-Panax notoginseng Preparations	CT	/	CT	Overall Effectiveness Rate; NIHSS	No	Excessive replication	Excessive replication	Excessive replication	Chinese
2021WangLD	/	/	4170	Multi-Panax notoginseng Preparations	CT	/	IVT,CT	mRS; Overall Effectiveness Rate; NIHSS; BI score	Yes	original	original	original	English
2024LiuYY	4.5h.	/	1856	Multi-Panax notoginseng Preparations	CT	/	IVT,CT	NIHSS; BI score	Yes	original	Conceptual replication	original	English
2023WangYT	/	Cerebral Infarction in Diabetic	1232	Multi-Panax notoginseng Preparations	CT	/	CT	NIHSS; BI score	No	Conceptual replication	Conceptual replication	Conceptual replication	Chinese

		Patients											
2023ShiXY	14d	/	7957	Multi-Panax notoginseng Preparations	CT	/	placebo,CT	mRS; BI score	Yes	original	original	original	English
2006LiKJ	30d	/	1460	Multi-Ginkgo biloba Preparations	others,CT	/	others,CT	Improvement in neurological function scores; Disability Rate; Mortality Rate; QoL; ADL Score	No	benchmark	benchmark	benchmark	Chinese
2015WangL	/	/	2292	Multi-Ginkgo biloba Preparations	CT	/	CT	Overall Effectiveness Rate; Improvement in neurological function scores;	No	Conceptual replication	Conceptual replication	Conceptual replication	Chinese
2015XuJY	14d	/	320	Multi-Ginkgo biloba Preparations	CT	/	CT	Overall Effectiveness Rate; Improvement in neurological function scores;	No	Conceptual replication	Conceptual replication	Conceptual replication	Chinese
2015QinSC	/	/	2292	Multi-Ginkgo biloba Preparations	CT	/	CT	Overall Effectiveness Rate; Improvement in neurological function scores;	No	Conceptual replication	Conceptual replication	Conceptual replication	Chinese
2017DongWS	72h	/	1045	Multi-Ginkgo biloba Preparations	CT	/	CT	Overall Effectiveness Rate;	No	Conceptual replication	Conceptual replication	Conceptual replication	Chinese
2018WangYS	/	/	1760	Multi-Ginkgo biloba Preparations	CT	/	CT	Overall Effectiveness Rate; NIHSS; BI score	No	Excessive replication	Conceptual replication	Conceptual replication	Chinese

2021ZhaoS	/	/	1259	Multi-Ginkgo biloba Preparations	CT	/	CT	NIHSS; BI score; Overall Effectiveness Rate;	No	Conceptual replication	Conceptual replication	Conceptual replication	English
2022LiTT	/	/	3907	Multi-Ginkgo biloba Preparations	CT	/	CT	NIHSS; mRS;	No	Conceptual replication	Conceptual replication	Conceptual replication	English
2025HuYQ	72h	/	6111	Multi-Ginkgo biloba Preparations	IVT,CT	/	IVT,CT	mRS; NIHSS; Overall Effectiveness Rate;	No	Conceptual replication	Conceptual replication	Conceptual replication	Chinese
2012MaLH	30d	/	2650	Multi-Ginkgo biloba Preparations	others,CT	/	others,CT	Overall Effectiveness Rate; Improvement in neurological function scores;	Yes	original	original	original	Chinese
2020ChongPZ	/	/	1466	Multi-Ginkgo biloba Preparations	CT	/	CT	All-CauseMortality; NIHSS; BI score; mRs; CognitiveFunction	Yes	original	original	original	English
2013XiaW	/	/	944	Sanqitongshu capsule	CT	/	CT	Improvement in neurological function scores	No	benchmark	benchmark	benchmark	Chinese
2018ChenX	/	/	981	Sanqitongshu capsule	CT	2-12w	CT	Overall Effectiveness Rate; Improvement in neurological function scores;	No	Excessive replication	Excessive replication	Excessive replication	Chinese
2012XiBC	/	/	726	Shuxuening injection	others,CT	/	others,CT	Overall Effectiveness Rate;	No	benchmark	benchmark	benchmark	Chinese
2014LiT	/	/	1798	Shuxuening injection	Edaravone,CT	/	Edaravone,CT	Overall Effectiveness Rate; Improvement in neurological function scores	No	Conceptual replication	Conceptual replication	Conceptual replication	Chinese
2023LiLD	/	/	1607	Shuxuening injection	Edaravone,CT	/	placebo,Edaravone,CT	NIHSS; NDSscore; ADLScore	No	Conceptual replication	Conceptual replication	Conceptual replication	English

2014HuJH	/	/	NA	Shuxuening injection	others,CT	/	others,CT	Overall Effectiveness Rate; Improvement in neurological function scores;	No	Excessive replication	Excessive replication	Excessive replication	Chinese
2024ZhanJ	14d	/	12401	Shuxuening injection	CT	/	CT	NIHSS; BI score	Yes	original	original	original	English
2024LiXK	/	/	1482	Xuesaitong softcapsule	others,CT	2w	others,CT	NIHSS;	No	Conceptual replication	Conceptual replication	Conceptual replication	Chinese
2022GengHJ	/	/	827	Xuesaitong softcapsule	CT	/	CT	Overall Effectiveness Rate; NIHSS; CSSscore;	No	Excessive replication	Excessive replication	Excessive replication	English
2022FengCN	/	/	1973	Xuesaitong softcapsule	others,CT	/	others,CT	Overall Effectiveness Rate; NIHSS; BI score	No	Excessive replication	Excessive replication	Excessive replication	Chinese
2022LiJQ	/	/	1235	Xuesaitong softcapsule	others,CT	/	others,CT	Overall Effectiveness Rate;	No	Conceptual replication	Excessive replication	Excessive replication	Chinese
2022GaoYJ	/	/	1942	Xuesaitong softcapsule	CT	/	CT	Overall Effectiveness Rate; BI score; CSSscore;	No	benchmark	benchmark	benchmark	English
2009DingX	/	/	NA	Xuesaitong injection	others,CT	/	others,CT	Overall Effectiveness Rate;	No	Conceptual replication	Conceptual replication	Conceptual replication	Chinese
2014ZhengCJ	/	/	1075	Xuesaitong injection	Ozagrel,CT	/	Ozagrel,CT	Overall Effectiveness Rate; Recovery Rate	No	Conceptual replication	Conceptual replication	Conceptual replication	Chinese
2019DuanXJ	/	/	2101	Xuesaitong injection	CT	/	salvia miltiorrhiza injection,CT	Overall Effectiveness Rate; Improvement in neurological function scores;	No	Conceptual replication	Excessive replication	Conceptual replication	English
2019LiuNN	/	/	1000	Xuesaitong injection	Ozagrel,CT	/	Ozagrel,CT	Overall Effectiveness Rate; NIHSS;	No	Conceptual replication	Conceptual replication	Conceptual replication	Chinese

2021YanMY	/	/	834	Xuesaitong injection	Edaravone,CT	/	Edaravone,CT	/	No	Conceptual replication	Conceptual replication	Conceptual replication	Chinese
2012TianCJ	30d	/	1441	Xuesaitong injection	others,CT	/	others,CT	Overall Effectiveness Rate; Improvement in neurological function scores	No	Excessive replication	Excessive replication	Excessive replication	Chinese
2014ZhangY	/	/	2295	Xuesaitong injection	CT	/	CT	NIHSS; mRs	No	Excessive replication	Excessive replication	Excessive replication	Chinese
2017TianP	7d	/	1354	Xuesaitong injection	CT	/	placebo,CT	Overall Effectiveness Rate; Improvement in neurological function scores	No	Excessive replication	Excessive replication	Excessive replication	Chinese
2021FengL	/	/	1769	Xuesaitong injection	CT	/	CT	Overall Effectiveness Rate; NIHSS;	No	Excessive replication	Excessive replication	Excessive replication	English
2023FengH	/	/	1843	Xuesaitong injection	CT	/	CT	Overall Effectiveness Rate;	No	Excessive replication	Excessive replication	Excessive replication	Chinese
2015ZhangXM	3d	TCM	2196	Xuesaitong injection	CT	/	CT	Overall Effectiveness Rate; Improvement in neurological function scores; BI score	Yes	original	original	original	English
2020SunXY	/	/	1128	Multi-Panax notoginseng Preparations	CT	/	CT	NIHSS;	Yes	original	original	original	Chinese
2016WangQ	72h	/	2903	Xuesaitong injection	others,CT	/	others,CT	NIHSS; ALD Score; Overall Effectiveness Rate; Improvement in neurological function scores;	Yes	benchmark	benchmark	benchmark	Chinese

2017WuFB	/	/	640	Xuesaitong injection	Butylphthalide,CT	/	Butylphthalide,CT	Overall Effectiveness Rate; Improvement in neurological function scores;	No	Conceptual replication	Conceptual replication	Conceptual replication	Chinese
2017ZhaoMR	72h	<80 years old	1342	Xueshuantong injection	fibrinolytic alone	/	fibrinolytic alone	Overall Effectiveness Rate; Recovery Rate	No	Conceptual replication	Conceptual replication	Conceptual replication	Chinese
2012LiHT	/	/	NA	Xueshuantong injection	others,CT	/	others,CT	Overall Effectiveness Rate;	No	Excessive replication	Excessive replication	Excessive replication	Chinese
2013ChenJ	/	/	1177	Xueshuantong injection	others,CT	/	others,CT	Overall Effectiveness Rate;	No	Excessive replication	Excessive replication	Excessive replication	Chinese
2018ChengMZ	/	/	2677	Xueshuantong injection	Edaravone,CT	/	Edaravone,CT	NIHSS; ESS; CSS; BI Score	No	Excessive replication	Excessive replication	Excessive replication	Chinese
2021RenFQ	/	/	1803	Xueshuantong injection	others,CT	/	others,CT	/	No	Excessive replication	Excessive replication	Excessive replication	Chinese
2023SongGL	/	/	5916	Xueshuantong injection	others,CT	/	others,CT	Overall Effectiveness Rate; NIHSS; BI score; ADL score	No	Excessive replication	Excessive replication	Excessive replication	Chinese
2008NiSQ	72h	/	2918	Yinxingdamo injection	others,CT	≥14d	others,CT	Overall Effectiveness Rate; Improvement in neurological function scores;	No	benchmark	benchmark	benchmark	Chinese
2013YuZW	/	/	1302	Yinxin Damo Injection	batroxobin	/	CT	Overall Effectiveness Rate; Improvement in neurological function scores; NIHSS; BI score;	No	Conceptual replication	Conceptual replication	Conceptual replication	Chinese

2016RenDQ	72h	/	NA	Yinxin Damo Injection	CT	/	salvia miltiorrhiza injection,	Overall Effectiveness Rate; Improvement in neurological function scores;	No	Conceptual replication	Excessive replication	Conceptual replication	Chinese
2019XueP	/	Articles involving more than 30 ischemic stroke patients	3182	Yinxin Damo Injection	CT	/	CT	NIHSS; BI score;	No	Conceptual replication	Conceptual replication	Conceptual replication	English
2018WangQ	/	/	1687	Ginkgo Diterpene Lactone Meglumine Injection	others, CT	/	others, CT	Overall Effectiveness Rate; Improvement in neurological function scores;	No	benchmark	benchmark	benchmark	Chinese
2019JinFH	/	Phase II, III, or IV RCTs	1129	Ginkgo Diterpene Lactone Meglumine Injection	others, CT	/	others, CT	Overall Effectiveness Rate; NIHSS; ADL score	No	Conceptual replication	Conceptual replication	Conceptual replication	Chinese
2021ZhangLL	/	/	2332	Ginkgo Diterpene Lactone Meglumine Injection	CT	/	CT	Overall Effectiveness Rate; NIHSS; BI score;	No	Conceptual replication	Conceptual replication	Conceptual replication	Chinese
2022ZhaoH	/	/	2389	Ginkgo Diterpene Lactone Meglumine Injection	IVT,CT	/	IVT,CT	Overall Effectiveness Rate; Improvement in neurological function scores;	No	Conceptual replication	Conceptual replication	Conceptual replication	English
2023YanMY	/	/	1636	Ginkgo Diterpene Lactone Meglumine Injection	Edaravone,CT	/	Edaravone,CT	Overall Effectiveness Rate; NIHSS; BI score; ADL score; mRS	No	Conceptual replication	Conceptual replication	Conceptual replication	English

2025WangLD	/	/	4041	Ginkgo Biloba extract	CT	/	CT	NIHSS; ADL score; mRS	Yes	Conceptual replication	Excessive replication	Conceptual replication	English
2025XuH	/	/	2195	Ginkgo Diterpene Lactone Meglumine Injection	Edaravone, CT	/	Edaravone, CT	Overall Effectiveness Rate; NIHSS; ADL score; mRS	No	Conceptual replication	Conceptual replication	Conceptual replication	English
2021LuoH	4.5h	/	765	Ginkgolide injection	IVT, CT	/	IVT, CT	Overall Effectiveness Rate; NIHSS; SF-36 score; mRS; BI score	No	benchmark	benchmark	benchmark	Chinese
2021MengTT	/	/	2059	Ginkgolide injection	CT	/	others, CT	Overall Effectiveness Rate; All-Cause Mortality; NIHSS; BI score; NDS score	No	Conceptual replication	Conceptual replication	Conceptual replication	Chinese
2021GuanYJ	/	/	1615	Ginkgo Ketone Esters	others, CT	/	others, CT	Overall Effectiveness Rate; NIHSS; ADL; Barthel ; mRS ; MMSE; MOCA.	No	benchmark	benchmark	benchmark	Chinese
2021MengTT	/	/	886	Ginkgo biloba leaf tablets	others, CT	/	others, CT	Overall Effectiveness Rate; NIHSS; NDS;	No	benchmark	benchmark	benchmark	Chinese
2024ChenZW	1m	/	2851	Ginkgo biloba leaf tablets	others, CT	/	others, CT	Overall Effectiveness Rate; Improvement in neurological function scores; Disability Rate;	No	Excessive replication	Excessive replication	Excessive replication	Chinese
2020JiHJ	/	/	1829	Ginkgo biloba leaf extract injection	CT	/	placebo, CT	All-cause mortality; BI scores; NIHSS; NFD Score	No	Conceptual replication	Conceptual replication	Conceptual replication	English
2017TanD	/	/	2364	Ginkgo biloba leaf extract injection	CT	/	Salvia Miltiorrhiza Extracts, CT	Overall Effectiveness Rate; NFD Score	Yes	original	original	original	Chinese
